# Patient-Accessible Electronic Health Records and Information Practices in Mental Health Care Contexts: Scoping Review

**DOI:** 10.2196/54973

**Published:** 2025-02-07

**Authors:** Timothy Kariotis, Megan Prictor, Kathleen Gray, Shanton Chang

**Affiliations:** 1 Faculty of Engineering and Information Technology University of Melbourne Carlton Australia; 2 Melbourne Law School University of Melbourne Melbourne Australia; 3 Centre for Digital Transformation of Health University of Melbourne Melbourne Australia

**Keywords:** patient-accessible electronic health records, patient portals, psychiatry, mental health, health informatics, mental illness, scoping review

## Abstract

**Background:**

Patients are increasingly being provided with access to their electronic health records. However, in mental health care contexts, concerns have been raised due to a perception that such access would pose risks to patients, third parties, and the therapeutic relationship. These perceived risks may affect the information practices of health care professionals (HCPs) and patients, such as how they document, share, and use information in mental health care services with a patient-accessible electronic health record (PAEHR). Although there is growing research interest in PAEHRs, no study has specifically examined how they impact information practices. Understanding the impacts on information practices may help explain other outcomes of implementing PAEHRs and inform their design.

**Objective:**

This scoping review aimed to explore the research on PAEHRs in mental health care contexts and how PAEHRs affect information practices of HCPs and patients in this context.

**Methods:**

A scoping review was considered the most appropriate method due to the relatively recent adoption of PAEHRs in mental health care contexts and the heterogeneous nature of the evidence base. A comprehensive search of electronic databases was conducted for original empirical studies that described the use of PAEHRs or associated systems in mental health care contexts. One author reviewed all full texts, with 3 other authors reviewing a subset of studies. The study characteristics and findings were documented, and a thematic synthesis and textual narrative analysis were used to develop descriptive and analytical themes that addressed the research questions.

**Results:**

A total of 66 studies were considered eligible and included in the analysis. The impact of PAEHRs on information practices in mental health care contexts included the following: (1) they may change how HCPs document patient information, including a reduction in detail and a focus on information perceived by HCPs as objective rather than subjective; (2) they may negatively impact workflows due to changes in documentation practices and limited guidance for HCPs on how to use PAEHRs; and (3) they may contribute to improved communication between HCPs and patients, including constructive disagreements regarding what is documented in the health record. The changes to HCP information practices were influenced by a concern for the therapeutic relationship and patient safety. Furthermore, PAEHRs supported new information practices for patients, such as using their PAEHR to prepare for clinical encounters.

**Conclusions:**

We have identified several ways in which PAEHRs shape the information practices of HCPs and patients in the mental health context. These findings can inform the design of PAEHRs to promote information practices that contribute to improving the quality of mental health care. Further research is necessary to understand how changes in information practices due to the implementation of PAEHRs impact clinical outcomes and patient experiences of care.

## Introduction

### Background

Patients in many high-income countries, such as Australia, the United States, the United Kingdom, France, and the Netherlands, have a legal right to request access to their health records [1,2]. Traditionally, patients have had to apply for a copy of their paper-based health records, which can hinder easy access and efficient use of their health information [3]. There is a growing movement to make it easier for patients to access their health information by providing electronic access to their health records. This shift toward online access has been enabled by the widespread adoption of electronic health records (EHRs) [2,4].

Providing patients with access to their health records can improve their disease management, self-care, and understanding of their health conditions [5], while facilitating more productive discussions between health care professional (HCPs) and patients [5-7]. Mental health care contexts have been slower to adopt patient-accessible EHRs (PAEHRs), and in some cases limitations have been placed on patients with mental health conditions accessing their PAEHR [8-12]. There are various concerns regarding the use of PAEHRs in mental health care contexts, including the impact on third parties, such as relatives whose information may be documented in the record and the risk that patients will experience distress from reading their PAEHR [11,13].

The digitalization of patient health records has impacted the information practices of HCPs and patients, changing the way they seek, document, and share information [14]. We use the concept of information practices to capture how information activities, such as documentation, are situated in and shaped by specific contexts [15]. Østensen et al [16] defined information practice as “a socially constructed practice that determines how information is produced, organized, disseminated, distributed, reproduced and circulated in the community, and which specific types of information are legitimized.” The concept of information practice is used in this study because it encourages consideration of factors unique to mental health care contexts, such as stigma and risk management, which may shape HCPs’ and patients’ information activities [17,18].

Several technologies are available to provide patients with access to parts or all of their health record, including electronic personal health records (ePHRs) and patient portals. A range of initiatives, such as OpenNotes and Blue Button, implemented predominantly in the United States and across several Nordic countries, have promoted immediate patient access to their EHRs via patient portals [19-21]. Both personal EHRs (pEHRs) and patient portals can be defined in comparison to EHRs, which are the digital clinical records held by a health service and accessible only by HCPs [22,23]. Patient portals provide patients with access to some parts of the EHR and a range of other functions, such as booking appointments [24]. In comparison, pEHRs are health records controlled by the patient, which may be linked to an EHR or stand-alone entities [25]. pEHRs may include a range of functions, such as capturing patient-generated data, such as biometric data from wearable devices, and providing access to educational material [26,27]. Beyond these simple definitions, there is substantial heterogeneity in the types, functions, architecture, and content of pEHRs and patient portals [26,28]. In this paper, because the focus is on patients’ online access to their health records, we use the term PAEHRs. This term captures the range of technologies that give people complete or partial access to the information contained in their EHR. Other terms may be mentioned in this paper when referring to results from specific studies.

Several recent reviews have explored the use of PAEHRs [29-32], with 2 specifically in mental health contexts [9,24]. However, these studies have not considered the impact of PAEHRs on information practices and have usually focused solely on patient portals. Zhang et al [24] found that the factors for successful implementation and adoption of patient portals in mental health care contexts include education for patients, the perceived value of the portal by patients, how easy it was for patients to use the portal, attitudes of HCPs toward using the portal, and adequate staffing and staff training to support the use of the portal. Schwarz et al [9] found that patients reported various positive experiences of using patient portals, such as increased empowerment and trust in clinicians, while negative experiences were related to inaccuracies in their records or disrespectful language. HCPs reported more concerns than benefits, including increased documentation burden and possible harm from patients reading their records. These studies reflect the growing prevalence and interest in PAEHRs in mental health care contexts. However, research is needed to consider the impact of PAEHRs on information practices because changes to information practices, such as HCPs documenting less information, may impact the quality and safety of care provided in mental health care settings.

This paper builds on our previous scoping review where we found that EHRs promote standardized and formalized documentation practices that often conflict with the prevalent use of narrative free-text information in mental health care settings [15]. The review also found that HCPs had concerns about documenting sensitive information in EHRs due to the increased ease of access by other HCPs [15]. The previous scoping review suggests that changing what is documented, how it is documented, and who can access information in the health record may alter clinicians’ information practices. Other studies have found that adopting EHRs affects patients’ intention to disclose certain information [33,34]. Thus, it could be assumed that the introduction of PAEHRs may impact HCPs’ and patients’ information practices.

This review has the following objectives and research questions:

1. How do PAEHRs change the information practices of patients and HCPs in mental health care contexts?
2. What effects do changes in information practices have on other aspects of care, including the therapeutic relationship, risk management, information sharing, and patient experience?

### A Note on Language

In this study, we used the term *patient* when referring to people accessing and using mental health services. We chose this over other terms, such as client or service user, mainly because many of the PAEHR technologies reference “patients” in their name (eg, patient portal), and we wanted to avoid causing confusion by using another term. We acknowledge that the term *patient* can be considered disempowering and that the terminology in this space is not settled.

The title of this paper refers to *mental health care contexts*, which is used to capture the broad range of clinical and nonclinical services people may access when experiencing mental health issues [35].

## Methods

### Overview

Scoping reviews facilitate consideration of a broad range of evidence, methods, and study types in relatively new or heterogeneous research fields [36]. Because PAEHRs in mental health care contexts are relatively new and information practices have received limited consideration in the literature, we considered a scoping review an appropriate method to address the research objectives [37,38]. We were informed by the framework for scoping reviews by Arksey and O’Malley [36] and the relevant criteria outlined in the PRISMA-ScR (Preferred Reporting Items for Systematic Reviews and Meta-Analyses extension for Scoping Reviews) checklist (Multimedia Appendix 1) [39]. No protocol was published for this review.

### Study Selection

The inclusion and exclusion criteria are outlined in Textbox 1. We chose to include soon-to-be-implemented PAEHRs, which we defined as systems that had been announced or were planned for implementation, for 3 reasons. First, if we were to publish an updated review, it would provide us with the opportunity to compare pre- and postimplementation studies. Second, perceptions of a PAEHR may influence practices as much as using a PAEHR [40,41]. Third, in our initial reading of the literature, we noticed that often PAEHRs had low use by HCPs or had only been recently implemented. Thus, there may be little difference between perceptions in the lead-up to implementation and perceptions after implementation if the PAEHR has not been widely used.

**Inclusion and exclusion criteria.**

**Inclusion criteria**

- Articles describing original empirical research
- Research conducted in a mental health care context (eg, psychiatric hospital) or with participants who have a lived experience of mental illness (eg, patients in a primary health care context with a diagnosis of depression)
- Studies published up to September 2023
- Studies focused on the implementation of a system for allowing patients with mental health conditions to access their health record or about the perception of an imminent implementation of such a system or studies that sought input from people with involvement in the design, adoption, use, or evaluations of patient-accessible electronic health records

**Exclusion criteria**

- Studies with no methodology
- Studies with no full text in English
- Protocol papers, commentaries, or posters
- Studies exploring health care professionals’ or patients’ general perceptions about access to online mental health records
- Studies where the participants are children or adolescents, or the study is conducted in the youth mental health system
- Studies conducted in forensic mental health care settings
- Scoping reviews or systematic reviews

### Search Strategy

We searched Scopus, Embase, Web of Science, MEDLINE via PubMed, PsycINFO, and CINAHL using a combination of key terms summarized in Textbox 2. A search was also conducted on Google and ResearchGate. The search strategy was developed iteratively alongside further identification of key terms in the literature and hand searching reference lists of relevant studies. The initial search was undertaken in late 2018 and updated in December 2022, with new papers continually identified until September 2023, when the final draft was completed.

**Example search strategy used in Scopus.**

(TITLE-ABS-KEY (“patient portal” OR “open*notes” OR “personal electronic record” OR “personal electronic health record” OR “personal electronic medical record” OR “personal medical record” OR “personal health record” OR “pEHR” OR “pEMR” OR “patient*controlled personal health records” OR “shared medical record” OR “patient controlled record” OR “patient*held medical record” OR “patient*controlled journal” OR “patient*controlled electronic health record” OR “blue button” OR “health record portal” OR “consumer portal” OR “patient access to health record” OR “PHR” OR “ePHR” OR “patient accessible record” OR “patient*shared record” OR “patient*carried record” OR “patient*held record” OR “patient internet portal” OR “patient accessible electronic health record” OR “patient access to record”) AND TITLE-ABS-KEY (“psychiatr*” OR “mental illness” OR “mental health” OR “mental health services” OR “behavioral health*” OR “mental healthcare” OR “mental health care” OR “mental health nurs*” OR “schizophrenia” OR “bipolar” OR “depression” OR “anxiety” OR “personality disorder” OR “psychosocial”))

### Screening Process

After removing duplicates, TK screened the titles and abstracts against the inclusion criteria to identify relevant studies for full-text screening. TK reviewed all full-text articles using the inclusion and exclusion criteria. TK identified 23% (15/66) of the studies for which it was unclear whether they met the inclusion criteria. The other 3 authors (MP, KG, and SC) each reviewed one-third (5/15, 33%) of these studies to determine whether they should be included. The authors worked together to resolve disagreements and determine the final articles for inclusion in the review. There were a few disagreements regarding studies in which the use of the PAEHR was minimal, as it was part of a larger study or intervention. Furthermore, 23% (15/66) of the studies that did not clearly meet the inclusion criteria were included after a discussion between the authors.

### Analysis of Included Studies

Due to the breadth of the study designs and objectives, covering a range of qualitative and quantitative methods, we adopted a textual narrative and thematic synthesis approach, as has been done in similar studies [15,42,43]. The textual analysis involved tabulating study findings alongside study characteristics and conclusions in a spreadsheet. The study characteristics we planned to document included the country where the study took place, year of publication, study design and method, participant characteristics, research focus, type of PAEHR, implementation status, and functions of the PAEHR. TK extracted these data from each paper into a spreadsheet, which was discussed with the research team to ensure its completeness.

Using the thematic synthesis approach described by Thomas and Harden [44], we developed descriptive themes by coding both direct participant quotes and researcher interpretations from the qualitative studies into a mind map. Our research questions framed this coding process, in that we coded findings related to how HCPs and patients used the PAEHR to manage information, how the PAEHR changed how HCPs and patients managed information, and findings related to issues raised in the Introduction section of this paper. Related codes were clustered together to develop descriptive themes. We intended these descriptive themes to stay as close to the original findings as possible. We integrated the quantitative data extracted during the textual synthesis into the descriptive themes. Finally, using our review questions, we developed analytical themes that sought to consider the impact of the PAEHRs on the information practices of HCPs and patients. This process is necessarily subjective and relied on the researchers’ judgment and insights [44,45]. To ensure the validity of the themes, TK undertook the initial descriptive analysis before seeking feedback from the research team to ensure the themes aligned with their understanding of the included studies and research questions. Similarly, TK developed the analytical themes before seeking feedback from the research team.

## Results

### Overview

**Figure 1:**
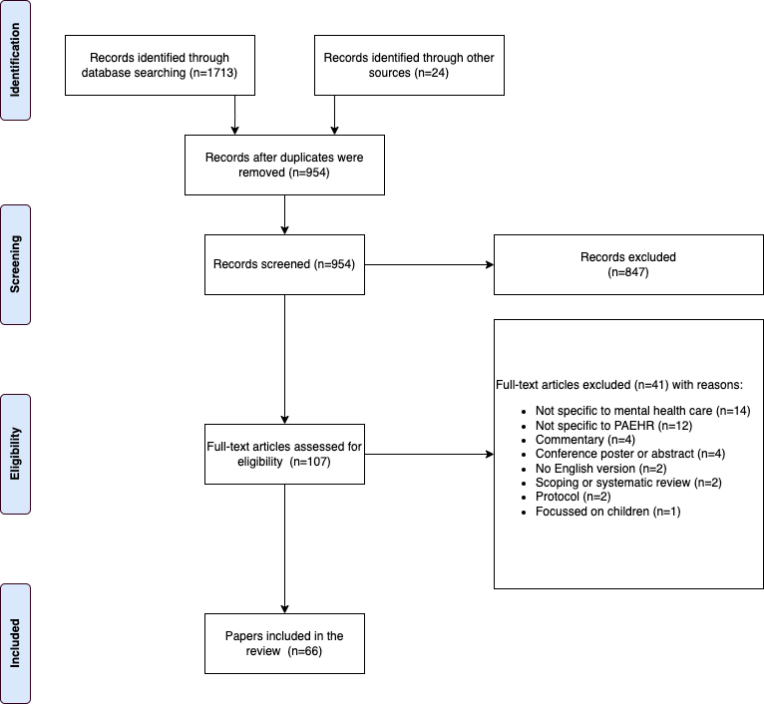
PRISMA (Preferred Reporting Items for Systematic Reviews and Meta-Analyses) diagram. PAEHR: patient-accessible electronic health record.

The search strategy identified 1713 studies. Another 24 studies were identified through hand searching and other sources, including Google and ResearchGate. After removing duplicates, of the 1737 studies, 54.92% (n=954) were identified for screening. After screening the titles and abstracts, of the 954 studies, 11.2% (n=107) met the inclusion criteria. Of the 107 articles reviewed in the full text, 38.3% (n=41) were excluded, and 61.7% (n=66) were included (Figure 1). Details of the included studies are outlined in Multimedia Appendix 2.

### Study Characteristics

The following sections report on the characteristics of the included studies that were identified in the textual synthesis.

#### Country

The top 3 countries where the studies were conducted were the United States (29/66, 44%), Canada (13/66, 20%), and Sweden (9/66, 14%). Table 1 provides a more detailed breakdown of the countries where the included studies were conducted. The main difference across countries was whether there was a national patient portal system, such as in Sweden, compared to a patient portal implemented across a specific service or set of services, such as the US Veterans Affairs My HealtheVet.

**Table 1:** Countries where the studies were conducted (N=66).

Countries	Studies, n (%)
United States	29 (44)
Canada	13 (20)
Sweden	9 (14)
Norway	4 (6)
The Netherlands	2 (3)
United Kingdom	3 (5)
Germany	2 (3)
New Zealand	1 (2)
Multiple countries	3 (5)

#### Year of Publication

**Figure 2:**
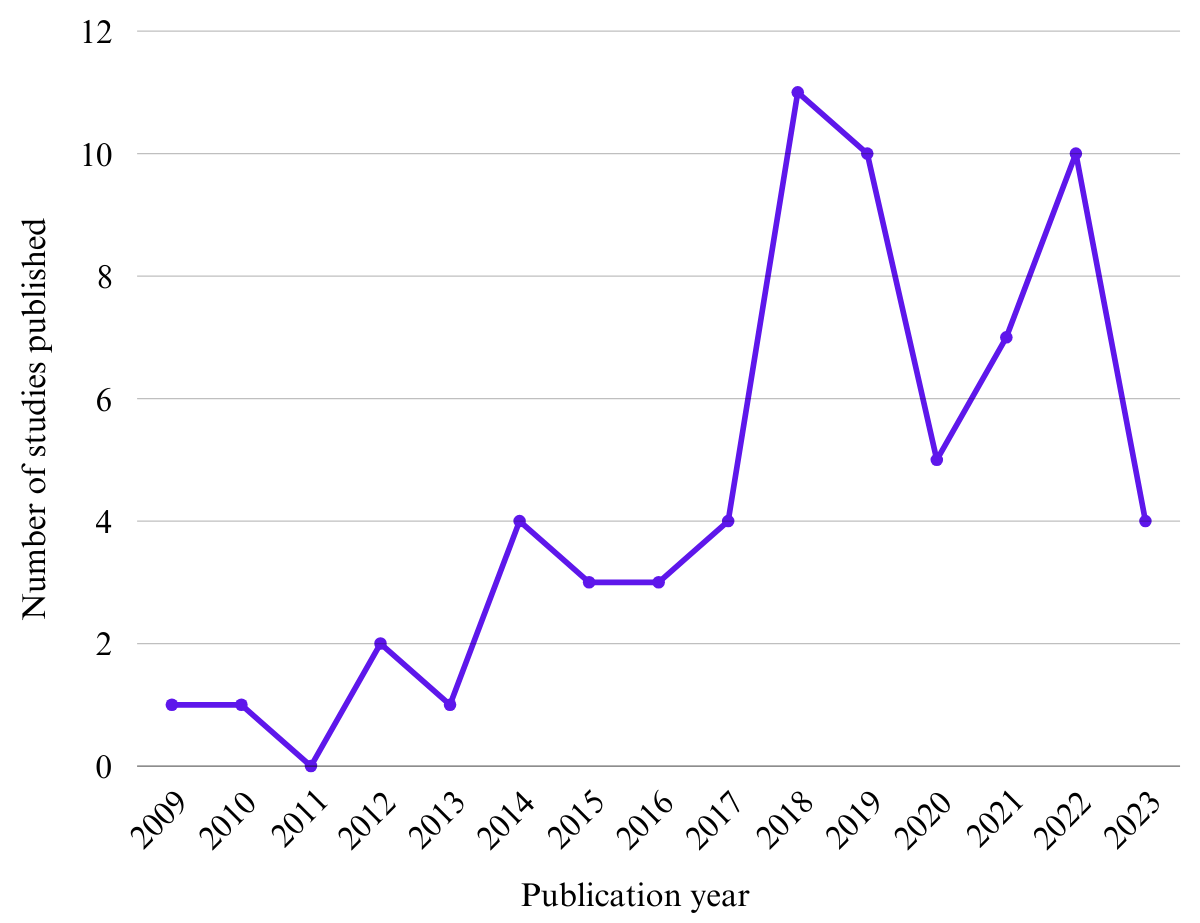
Increasing trend in publications on patient-accessible electronic health records in mental health care contexts.

The included studies were published in the period from 2009 to 2023 (the cutoff year for inclusion). The highest number of studies was published in 2018 (11/66, 17%), followed by 2019 (10/66, 15%) and 2022 (10/66, 15%). There is an overall upward trend of studies published on PAEHRs in mental health care contexts since 2009, with some fluctuations in recent years, as illustrated in Figure 2. Due to the varied level of detail reported in the included studies on the functions and technical features of the PAEHRs, we did not evaluate how PAEHRs have changed over time.

#### Study Design and Method

There was a mix of quantitative (29/66, 44%), qualitative (22/66, 33%), and mixed methods (15/66, 23%) approaches used in the included studies (Table 2). The main methods used were surveys (27/66, 41%), interviews (18/66, 27%), and interventions or trials involving a PAEHR (13/66, 20%).

**Table 2:** Study design and method (N=66).

Designs and methods		Studies, n (%)
**Study design**		
	Quantitative	29 (44)
	Qualitative	22 (33)
	Mixed methods	15 (23)
**Research methods^a^**		
	Survey	27 (41)
	Interview	18 (27)
	Focus group	7 (11)
	Interventions or trials involving a PAEHR^b^ (eg, randomized controlled trials)^c^	13 (20)
	Secondary data analysis	7 (11)
	Design	2 (3)
	Observation	1 (2)
	Document analysis	1 (2)

^a^Some studies included multiple methods and thus were counted twice.

^b^PAEHR: patient-accessible electronic health record.

^c^We did not report the specific methods of intervention studies; instead, we just categorized them as interventions.

#### Participants

Most studies (37/66, 56%) included only patients as participants, while 27% (18/66) of the studies included only HCPs, and 15% (10/66) of the studies included both patients and HCPs. Table 3 provides a more detailed breakdown of participants. Nurses were the most represented HCPs, followed by social workers, psychologists, psychiatrists, and physicians. Family members, carers, and peer workers were rarely involved as participants in the included studies.

**Table 3:** Participants in the included studies (N=66).

Participants		Studies, n (%)
**Study sample (high level)**		
	HCPs^a^	18 (27)
	Patients	37 (56)
	Both HCPs and patients	10 (15)
	Other or not specified	1 (2)
**Study sample (detailed)**		
	Patients	48 (73)
	Family and carers	3 (5)
	Peer workers	3 (5)
	Social workers	17 (26)
	Psychiatrists	14 (21)
	Psychologists	16 (24)
	Mental health nurses, nurse practitioners, nurses, and nurse assistants	19 (29)
	Physicians, doctors, general practitioners, and family physicians	14 (21)
	Allied health—not including psychologists (occupational therapists, counselors, and physiotherapists)	5 (8)
	Administrative staff	7 (11)

^a^HCP: health care professional.

#### Research Focus

We compared the aims of the included studies and grouped them according to similar topic areas, as outlined in Table 4. Most studies focused on the general experiences and perceptions of HCPs (18/66, 27%) and patients (13/66, 20%) toward a PAEHR. Several studies also explored specific clinical interventions or models of care using a PAEHR (12/66, 18%) and adoption and use rates across different demographic groups (11/66, 17%).

**Table 4:** Research focus (N=66).

Research focus	Studies, n (%)^a^
HCPs’^b^ experience and perspectives of PAEHR^c^	18 (27)
Patients’ experience and perspectives of PAEHR	13 (20)
Interventions using a PAEHR	12 (18)
Adoption and use of PAEHR by different patient groups	11 (17)
Design and usability of PAEHR for patients	7 (11)
Barriers and facilitators to patients using PAEHR	4 (6)
HCP or patient perspectives of the impact of PAEHR on therapeutic relationships	5 (8)
Exploring how education or training can support the use of PAEHR by an HCP or patient	3 (5)
Describing policies related to PAEHR	2 (3)
Exploring the impact of PAEHRs on transparency in the clinical encounter	2 (3)
Perceived benefits, harms, or risks of PAEHRs	2 (3)
Other (exploring errors in PAEHR, changing HCP practices, family or carer perceptions, reasons patients do not adopt PAEHR, perspectives on the needs of patients and clinicians when using OpenNotes, and impact of PAEHR on medication adherence)	6 (9)

^a^Some studies included multiple research aims and thus were counted twice.

^b^HCP: health care professional.

^c^PAEHR: patient-accessible electronic health record.

#### PAEHR System Used

There were several challenges in defining the PAEHR system used in each study. Most studies (26/66, 40%) discussed patient portals or OpenNotes generally without specifying a specific product or whether the system was a patient portal of ePHR (Table 5). Some studies discussed systems that appeared to be implemented at the national or health system level, although it was difficult to discern across studies if the same system was being discussed.

**Table 5:** Patient-accessible electronic health record (PAEHR) systems reported in the included studies (N=66).

Type of PAEHR	Patient portal or ePHR^a^ (categorized by author)	Studies, n (%)
Lawson Smart Record	ePHR	4 (6)
Veterans Affairs My HealtheVet	Patient portal	13 (20)
Patient Portal—general (including OpenNotes)	Patient portal	26 (40)
Swedish Journalen	Patient portal	8 (12)
Helsenorge.no [46-49]	Patient portal	4 (6)
ePHR—general	ePHR	6 (9)
My Health Locker	ePHR	2 (3)
EPIC MyChartPortal	Patient portal	1 (2)
My Medical Record	Patient portal	1 (2)
MyCare (Cerner patient portal)	Patient portal	1 (2)

^a^ePHR: electronic personal health record.

#### Implementation Status

Most studies (51/66, 77%) reported on a PAEHR that had been implemented (Table 6).

**Table 6:** Implementation status of patient-accessible electronic health record (PAEHR; N=66).

Implementation status of PAEHR	Studies, n (%)
Implemented PAEHR	51 (77)
Soon-to-be-implemented PAEHR	5 (8)
PAEHR being designed as part of the study	3 (5)
Not applicable (expert perspective)	2 (3)
Partial implementation, including if some participants were using or had access to the PAEHR	3 (5)
Pre-post implementation	1 (2)
Unclear	1 (2)

#### Functionality of PAEHR

The included studies did not provide enough information for a thorough analysis of the functions available across the different PAEHRs. Therefore, we only address specific elements of functionality related to the themes discussed in the results of the thematic synthesis discussed in the Thematic Results section.

### Study Quality

Due to the heterogeneity of the included studies and the lack of evaluation instruments that could be applied systematically across these studies, a quality evaluation was not deemed appropriate for this scoping review. It is optional for scoping reviews to evaluate the quality of the included studies [50]. One quality issue we identified during the review of the included studies was a lack of detail regarding the functionality and technical specifications of the PAEHRs, which Talmon et al [51] recommend should be reported in health informatics studies. We identified a lack of reporting on functionality and technical specifications in our previous scoping review of EHRs [15].

During the peer-review process, a reviewer identified that 4 articles were published in journals that they considered predatory. We concluded that publishing in a predatory journal is not necessarily an indication of the quality of the study, and thus, we decided to include these studies in the review.

### Thematic Results

#### Overview

In the following sections, we report on the findings of the thematic synthesis of the 66 included studies. The analysis led to the development of 8 themes and 12 subthemes. The themes are outlined in Table 7. Illustrative quotes for each theme are provided in Multimedia Appendix 3.

**Table 7:** Summary of themes.

Themes	Subthemes	
Theme 1: patient-accessible electronic health records (PAEHRs) change the purpose of the health record	—^a^	
Theme 2: health care professionals (HCPs) change their information practices to protect the therapeutic relationship and patient safety	Subtheme 2.1: PAEHRs introduce risks to the therapeutic relationship Subtheme 2.2: PAEHRs introduce risks to patient safety Subtheme 2.3: trust, transparency, and accuracy of information can mitigate the risks posed by the PAEHRs to the therapeutic relationship and patient safety Subtheme 2.4: PAEHRs offer opportunities to enhance accountability and communication between HCPs and patients regarding the documentation of information	
Theme 3: PAEHRs disrupt HCPs’ documentation practices	Subtheme 3.1: HCPs document less detailed information in PAEHRs, particularly relating to sensitive information Subtheme 3.2: PAEHRs require HCPs to document information that patients will understand Subtheme 3.3: PAEHRs require HCPs to take a person-centered approach when documenting potentially subjective information Subtheme 3.4: HCPs limit the documentation of uncertain information in PAEHRs Subtheme 3.5: HCPs may omit content or restrict access to PAEHRs	
Theme 4: PAEHRs introduce changes to HCPs’ information workflows	—	
Theme 5: HCPs require tailored training, education, and guidelines on documenting information in a PAEHR	—	
Theme 6: patients are empowered with new information practices if they are supported to use their PAEHR	Subtheme 6.1: patients need support to use their PAEHR Subtheme 6.2: PAEHRs must be easy for patients to navigate, access, and understand	
Theme 7: PAEHRs raise new concerns about information privacy and security for HCPs, patients, and third parties	Subtheme 7.1: PAEHRs pose challenges to managing third-party data Subtheme 7.2: PAEHRs pose challenges to managing third-party access to patient information	

^a^No subthemes.

#### Theme 1: PAEHRs Change the Purpose of the Health Record

HCPs perceived that the introduction of a PAEHR changed the purpose of the health record, with HCPs concerned by the increasing array of audiences they had to consider when documenting information [49,52-54]. This change was framed as a culture shift [55], with one HCP in the study by Denneson et al [53] describing it as “antithetical to the way that many of us have been, literally, trained and learned to think about our field.” HCPs thought the way information was documented previously, which assumed that only other HCPs would access it, was inappropriate for patients and would pose risks such as worry, offense, or distress for patients [47,49,52,56]. Conversely, there was a perception that writing only for the patients may reduce the clinical value of the EHR [57]. HCPs in several studies also perceived a shift in control over the health records due to the ability of patients to access their health records in real time via their PAEHR [49,56,57]. Although patients may have previously been able to request access to their records, clinicians described having had more control over what was released and when it was released [49,53]. HCPs also felt they had less control over what patients might do with the information in their PAEHR, such as sharing it with third parties [58].

#### Theme 2: HCPs Change Their Information Practices to Protect the Therapeutic Relationship and Patient Safety

##### Overview

HCPs were concerned that PAEHRs would damage the therapeutic relationship because patients may not understand what is documented in their PAEHR or experience distress from reading their PAEHR. Some HCPs and patients recognized that PAEHRs could enhance the therapeutic relationship through improved transparency and communication about what was documented. However, the perceived risks appeared to be significant enough to change HCPs’ information practices. These perceived risks are part of a broader perception, outlined in theme 1, that the purpose of the record changes when patients have access to it via a PAEHR and that this puts into question previous practices around what is documented, for whom, and for what purpose.

##### Subtheme 2.1: PAEHRs Introduce Risks to the Therapeutic Relationship

HCPs across several studies reported concerns that PAEHRs would negatively impact the therapeutic relationship due to patients not understanding what was written in their record or perceiving something written in their record to be a negative judgment about them [53,58-61]. This risk shaped many of the documentation changes described in theme 3. HCPs described a risk that patients might see a disconnect between what was written in their record and their in-person clinical experience [53]. For example, what HCPs document is not always the same information they would share with patients, such as clinical formulations [53]. HCPs in the study by van Rijt et al [58] perceived a risk that patients might feel “insulted, misinterpret the information given, or feel unheard,” which in turn may negatively impact a patient’s trust in the HCP. In comparison, patients in the included studies focused more on the benefits of the PAEHR for the therapeutic relationship, as considered in subthemes 2.3 and 2.4.

##### Subtheme 2.2: PAEHRs Introduce Risks to Patient Safety

HCPs across several studies reported a concern for patient safety, specifically that patients might find the PAEHR overwhelming and distressing or be triggered by reading their records [49,53,55,57-59,62]. For example, Turvey et al [62] reported that 55% (16/29) of HCPs in their study reported having patients who experienced significant distress after reading their records, while 29% (8/29) and 21% (6/29) of HCPs reported experiences of patients terminating treatment after accessing their records or reporting having engaged in “negative and/or self-destructive behavior toward themselves or others,” respectively. These results do not specify how many patients had negative experiences of reading their PAEHR. Rather these results suggest that HCPs are coming across examples of patients’ having negative experiences, which is shaping their perception of PAEHRs. These concerns could be considered through the lens of HCPs’ ethical need to keep patients safe, with HCPs in the study by Jonnergård et al [63] identifying “patient safety” as the most important issue to be addressed in implementing OpenNotes. In addition, there were some concerns that PAEHRs might lead to increased violent behavior by patients. However, studies that explored these concerns did not find evidence of increased violent behavior from the adoption of PAEHRs in mental health contexts [64,65].

Patients were concerned about the risk of being upset by access to their PAEHR or experiencing anxiety when thinking about what might be written in their PAEHR [57,66]. For example, 11% (4/37) of patients reported “sometimes experiencing significant distress” after reading their record in the study by Turvey et al [62]. Denneson et al [67] found in patient survey that 26.4% (47/178) of patients sometimes, and 8.4% (15/178) often or always, “experienced stress or worry after reading their record,” while 18% (32/178) of patients sometimes, and 8.4% (15/178) often or always, reported “feeling upset.” A common reason reported for patients feeling upset after accessing their PAEHR was because they made the patients’ problems seem smaller than they were [67]. Some patients in the study by Schwarz et al [68] also reported a fear of reading their records because of how confronting it may be to revisit the experiences they had shared with their HCPs during clinical sessions. Alternatively, accessing information in the PAEHR between appointments could reduce the anxiety of waiting to see the HCP [37]. Interestingly, the 1 study that compared patients with mental health diagnoses to patients without mental health diagnoses found no difference in perceptions, including the likelihood to report worry, toward viewing their records [69].

##### Subtheme 2.3: Trust, Transparency, and Accuracy of Information Can Mitigate the Risks Posed by the PAEHRs to the Therapeutic Relationship and Patient Safety

Trust and transparency mediated the potential negative impact of PAEHRs on the therapeutic relationship. Several studies found that PAEHRs could support the therapeutic relationship through improved transparency and trust [37,38,52,54,57,68], particularly when information in the PAEHR aligned with what was discussed in the clinical encounter [38,66,67]. For example, the study by Cromer et al [38] reported a negative impact on patients’ trust in HCPs when they perceived low transparency about what was documented and when there was outdated information, mistakes, details that did not align with their recollection, or missing information. Similarly, patients in the study by Pisciotta et al [61] reported that the quality of information documented in their PAEHR was important to patients, including accurate notes that were not copied and pasted from previous sessions. Schwarz et al [68] described this as a “trust, but verify” situation, where patients valued the opportunity to confirm that HCPs understood them and their needs. Accuracy was also important, as patients were worried that inaccurate records could negatively affect their future treatment [38].

##### Subtheme 2.4: PAEHRs Offer Opportunities to Enhance Accountability and Communication Between HCPs and Patients About What Is Documented

Communication between HCPs and patients about the PAEHR and what was being documented was identified as important for building trust and strengthening the therapeutic relationship [38,50,52,53,57,66,67,70]. HCPs in the study by Denneson et al [53] discussed the role of communication, disagreements, and listening as important to consider when documenting in the PAEHR. In the study by Cromer et al [38], patients spoke of trust being built when HCPs listened to them, approached the relationship as an equal, and focused on the patient’s strengths. Several studies suggested that communication about what was documented, even disagreements, could positively contribute to the therapeutic relationship [38,53,58,62]. Interestingly, Pisciotta et al [61] found that patients and HCPs avoided discussing health records for different reasons. HCPs were worried about patients challenging their records or requesting changes, and patients were worried about appearing difficult or offending the HCP. However, both HCPs and patients agreed that having a conversation about information before it is documented in the PAEHR is beneficial [61].

Although both HCPs and patients fear disagreements over what is documented in the PAEHR, disagreements could also provide an opportunity to improve accountability and communication [37,46,49,58,68,71]. Disagreements may come about due to legitimate errors in what was documented, a misunderstanding of what was discussed and documented, or an actual disagreement between the HCP and patient as to what they understood was true [46,49,52,53,62]. However, these disagreements could improve the PAEHR by addressing errors or misunderstandings and providing opportunities to discuss issues that were previously left unspoken [46,54,58,71]. Patients commonly identified errors in their PAEHR; in a national Swedish survey by Bärkås et al [72], half of the respondents (1586/3131, 50.56%) from mental health care contexts reported finding an error, while a third (1089/3131, 34.78%) reported finding an omission. In addition, Bärkås et al [72] found that patients in mental health care settings were more likely to identify errors and rate them as serious than patients in other health care settings. Some HCPs saw the PAEHR as a way to increase accountability by having a feedback loop where patients could review their health record and report inaccuracies, errors, or misinterpretations [53,54]. Knowing that patients may disagree with what is written in the PAEHR may motivate clinicians to proactively discuss what they are documenting with patients and consider patients’ needs when documenting information [53,61,62].

Although HCPs were concerned that some patients may try to dictate what should be written in their PAEHR and the care they receive [54], there was limited evidence of this occurring. For example, only 15.4% (~51/332) of HCPs in the study by Johansen et al [47] reported receiving patient feedback. However, this may be due to patients’ low awareness of the PAEHR, as outlined in theme 6.

#### Theme 3: PAEHRs Disrupt HCPs’ Documentation Practices

##### Overview

HCPs reported that giving patients access to their mental health records would lead to or had led to changes in how they documented information [47,49,53,57,58,61,65,73,74]. These changes were usually framed as an attempt to protect the therapeutic relationship from potential misunderstandings or to protect patients from distress caused by reading their health record. As outlined in theme 2, HCPs appear to take a risk-averse approach to PAEHRs due to a perception that patients may react negatively or experience distress from reading their PAEHR. However, HCPs also seem to recognize that certain ways of framing information about patients may trigger a negative reaction, disagreement, or distress. There was a lack of guidance, support, or training for HCPs on managing these risks when documenting information in a PAEHR [53,54,56,75].

##### Subtheme 3.1: HCPs Document Less Detailed Information in PAEHRs, Particularly Relating to Sensitive Information

Clinicians addressed perceived risks posed by the PAEHR by omitting or “watering down” information that the patient might perceive negatively [53,57-59,65,70,76]. Petersson et al [65], in exploring the adoption of OpenNotes in Swedish Psychiatric Care settings, found that 22% (147/667) and 17.7% (117/662) of HCPs surveyed were less candid in their documentation and took more time to edit notes, respectively. Similarly, 63% (127/~208) of HCPs in the study by Dobscha et al [59] reported that they are or plan to be less detailed in their documentation. One reason reported for including less detail was a perception that more detailed notes increased the potential for misinterpretation and disagreements [61]. An HCP in the study by Zanaboni et al [49] described it as not writing “things that you can keep to yourself,” such as longer hypotheses. However, some HCPs considered that the benefit of documenting complete information outweighed the risks and that any harm could be repaired through discussions with the patient [49]. Patients preferred detailed notes that provided a complete picture of their appointment and mental health, as they perceived the health record to be a reflection of the HCPs’ understanding of them [61].

HCPs and patients agreed that sensitive information required careful management in how it was documented in PAEHRs [54,58,61,65,71,74-78]. Sensitive information was defined broadly, including information that patients may not want to be shared beyond the clinical encounter, information that may be triggering or distressing for the patient, information that might be stigmatizing, and information related to the risk of self-harm or suicide [54,58,71,75,78,79]. It was common for studies to refer to mental health information generally as sensitive information. Some clinicians reported excluding specific details from the PAEHR that could be triggering, traumatic, or cause harm to patients [58,71,76]. For example, participants in the study by Kassam et al [54] discussed not documenting specific instances of physical abuse reported by patients experiencing domestic violence. Instead, the HCPs may imply domestic violence in the record by focusing on the safety planning they had discussed with the patient. There was agreement across HCPs and patients in the study by Pisciotta et al [61] that traumatic information should not be recorded in detail in the PAEHR.

HCPs identified various risks to documenting less detailed information, including that it might impact the clinical value of the EHR, particularly if the information is not detailed enough for other HCPs [54,58,62]. HCPs in the study by van Rijt et al [58] considered that they are responsible for what is documented and for what is not documented, as well as the impacts these documentation choices have on the patient. Patients may also rely on their health record to access certain services or support, which may require detailed information [61,67].

##### Subtheme 3.2: PAEHRs Require HCPs to Document Information That Patients Will Understand

One way that HCPs changed their documentation was to reduce or reframe the use of medical terminology or jargon that patients might not understand [54,55,61,71,80]. This change was something that both HCPs reported doing [49,54,61,66,71] and that patients wanted HCPs to do [61,66,68]. In one of the few reported examples of HCPs receiving guidelines for using a PAEHR, the guidelines recommended that HCPs limit the use of abbreviations, medical language, or euphemisms, even if they may be commonly used in the medical profession [81]. However, medical terminology may also be used by HCPs to communicate with other health HCPs or to disguise information from the patient [49,56]. Another approach to managing medical terminology was proactively discussing it with the patients to manage any potential confusion the terms might cause [54].

##### Subtheme 3.3: PAEHRs Require HCPs to Take a Person-Centered Approach When Documenting Potentially Subjective Information

HCPs reported that they changed their documentation to be more objective and less open for interpretation in the presence of a PAEHR [58,71,76]. For some HCPs, this change was related to a concern that information in the record may offend patients [47,52]. For example, HCPs discussed the challenge of documenting a patient’s behavioral or physical presentation, which the patient could perceive as a subjective judgment [54]. Participants in the study by Blease et al [71] recommended that clinicians avoid demeaning, embarrassing, or stigmatizing terms such as “patient complains of.” HCPs were conscious of medical terms that had stigmatized social meanings, such as “psychotic,” that could lead to misunderstandings with patients [62,76]. This concern meant that HCPs might not document information related to the clinician-patient relationship, the patient’s personality, or certain symptoms such as paranoia and may instead provide less detailed objective information [54,70]. However, it was recognized that some mental health assessments require subjective input from the HCP and that not including this information in the EHR might impact future episodes of care and cause confusion among the care team [54].

HCPs across several studies acknowledged that PAEHRs encourage them to write more person-centered and empathetic information [53,54,61]. In the study by Kassam et al [54], HCPs reported that the PAEHR made them conscious of language that “may be perceived as judgmental and demoralizing.” Recognizing that a negative tone could come across as judgmental, some clinicians sought to write in a tone that conveyed empathy [61]. This need formed part of a broader consideration that HCPs should not just highlight the deficits and issues in the record but also the strengths and unique attributes of the patient [61]. Some HCPs sought to document more information on patients’ strengths and recovery when using a PAEHR [54,61]. Patients have reported wanting HCPs to include such information in their record [61]. There was an acknowledgment by HCPs that while some information may be perceived as offensive, other information was explicitly disrespectful, and PAEHRs might help minimize such language [70].

It was important to patients that that the information documented in their PAEHR was respectful and empathetic. Participants in the studies by Cromer et al [38] and Fagerlund et al [46] wanted their health record to reflect them as a whole person rather than just containing “surface level observations.” Patients thought that HCPs could show respect by being mindful of their tone and words that may appear as judging or labeling [61]. In the study by Bärkås et al [72], a third of patients in a national survey reported feeling offended by something written in their PAEHR.

##### Subtheme 3.4: HCPs Limit the Documentation of Uncertain Information in PAEHRs

HCPs discussed the challenge of documenting uncertain or unfinalized information in the PAEHR, such as diagnostic hypotheses or information that had not been confirmed with the patient [49,56-58,71,78]. For example, HCP might avoid documenting the assessment process leading to a final diagnosis or treatment plan because a patient might read those initial notes without knowing that it is not a final decision [49,53,56]. HCPs may withhold documenting suspected diagnoses due to the risk of upsetting or alarming the patient [54] or wait to discuss the diagnosis face-to-face with the patient before documenting it [49]. Some studies reported cases of patients discovering a diagnosis in their record that had not been discussed with them previously [38,61]. However, documenting uncertain information could play an important role in collaborative care arrangements and handovers, where multiple HCPs contribute to an assessment and need access to this information [56]. This information may also form an important part of the clinician’s work to assess a patient and formulate a diagnosis or treatment plan [57].

##### Subtheme 3.5: HCPs May Omit Content or Restrict Access to PAEHRs

HCPs discussed various scenarios where information might be withheld from patients or omitted from the PAEHR [47,49,52,56,58,59,62,70,78]. These scenarios included where patients were experiencing delusions or suicidal thoughts that could be exacerbated by access to their records [54] or where a patient’s knowledge of a treatment plan in advance, particularly involutory treatment, may hinder the provision of that treatment [58]. This practice may occur either through actively omitting information from the PAEHR, maintaining information in a separate informal record not sanctioned by the health service, or recording the information in a section of the PAEHR that patients cannot access. Some PAEHRs offered functionality to restrict or hide information from patients [47,53,60]. Some PAEHRs provided functionality to limit patient access to information, including allowing for a delay to be implemented before patients could view information [49,78,82], allowing only “signed” notes that an HCP had validated to be viewed by patients [47,52,78,82], or providing specific templates where HCPs could document sensitive information that patients could not view [65,74,82]. Some HCPs in the study by Zanaboni et al [49] mentioned keeping a physical folder with information that had not yet been included in the EHR. The risk of such unsanctioned records is that the information may be lost if not entered into the EHR or inaccessible by other HCPs [56]. HCPs discussed the need for discretion in determining when to withhold information from patients, particularly when there is a justified reason for doing so [54]. Other HCPs thought that patients should have access to all their health information and that all patients should have equal access to their PAEHR [53], a view also held by some patients [83]. Limiting patient access to information, particularly mental health information, could perpetuate stigma and distrust of services [54].

HCPs across several studies identified certain patients that they considered at increased risk of negative outcomes from accessing their PAEHR [47,49,59,65,71,76]. The groups reported were those with psychosis, paranoia, high levels of suspicion, or personality disorders [49,65,71]. In a pilot study of OpenNotes by Peck et al [76], where HCPs could choose which patients to enroll, HCPs reported choosing patients based on the severity of their illness, length of time in treatment, and psychiatric diagnosis. Similarly, in the study by Zanaboni et al [49], HCPs in an outpatient setting discussed how they do not treat “the most challenging patients.” HCPs in the study by Kassam et al [54] reported that their documentation practices may change with specific patients in mind, including their diagnosis and clinical program. HCPs may also consider the context, such as whether patient access during a crisis may negatively shape their engagement with mental health care services, particularly planned involuntary treatment [58]. Although HCPs across several studies believed that a certain subgroup of patients, either due to diagnosis or current symptoms, may be at greater risk of harm from accessing their health record, there seemed to be a lack of guidance or support for making these decisions. Thus, it may be that the available evidence does not accurately reflect the experiences of certain groups of patients if HCPs are actively not encouraging certain patients to use their PAEHR, or because studies are not being undertaken in settings with patients who have more complex conditions. This finding that HCPs may avoid involving certain patients in using their PAEHR may also explain why, in some studies, patients with certain diagnoses had lower odds of being registered for a PAEHR [84-87].

#### Theme 4: PAEHRs Introduce Changes to HCPs’ Information Workflows

HCPs across several studies reported that PAEHRs would or had increased their workload due to changes in documenting information, answering patient questions, and reassuring patients [10,47,49,52,55,58,59,65,68,80]. However, in other studies, HCPs did not report any change to their workflow or workload [49,52,59,65,71] or suggested that changes in documentation may only require more work initially to adapt their documentation style [54]. For example, only 14.5% (86/594) of HCPs in the study by Petersson et al [65] reported that appointments took longer, and 18% (106/588) reported spending more time answering patient questions. The variation in the impact of PAEHRs on HCP workflows may be related to how the PAEHR was implemented and the level of support provided to HCPs and patients. One workflow change reported was the order of communication and documentation of information [49]. As previously discussed in subtheme 3.5, HCPs across several studies had previously used the health record to document preliminary information. The adoption of a PAEHR led HCPs to document this preliminary information elsewhere or not at all or to have earlier discussions with the patient about this information [49,56-58,71,78]. Another workflow issue raised by HCPs in the study by van Rijt et al [58] was the time required to explain what is written in the PAEHR and update the PAEHR if a patient disagreed with what was documented. In some cases, workflow issues were related to technical issues with the PAEHR, such as slow sign-in or troubleshooting technical issues for patients [88]. Interestingly, patients in the study by Schwarz et al [68] were concerned that the PAEHR might undermine the trust established with their HCP partly due to an increased HCP workload.

#### Theme 5: HCPs Require Tailored Training, Education, and Guidelines Documenting Information in a PAEHR

There was a clear lack of support and guidelines for HCPs across the included studies [49,53,75]. As outlined in theme 1, PAEHRs were viewed by HCPs as contrary to how they were trained. Several studies discussed the need for HCPs to receive training and support in using and promoting PAEHRs, such as how to write notes appropriately for patients [53,61,73,75,89,90]. Some of the issues identified as requiring guidance included describing sensitive matters and writing information “that may potentially be perceived in a negative light in a way that is clinically appropriate and respectful” [71]. When guidance was unavailable, HCPs took a risk-averse approach by adopting the information practices outlined in theme 3, such as reducing the level of detail documented about patients.

The few studies that included training for HCPs found mixed results regarding its effectiveness. Dobscha et al [73], reporting on a study of web-based education programs for HCPs using OpenNotes, found that after training, clinicians were better able to communicate, educate, and advise patients on accessing and reading their notes. The training also reduced HCPs’ concerns regarding the potential negative outcomes of OpenNotes. However, HCPs in the study by Zanaboni et al [49] who also completed internet-based learning modules discussed how they would like more formal training in using the PAEHR, as their memories of the online training were vague. HCPs in the study by Jonnergård et al [63] identified that HCPs preferred to receive information about OpenNotes via channels that allowed for dialogue and rich information, such as workplace meetings.

#### Theme 6: Patients Are Empowered With New Information Practices if They Are Supported to Use Their PAEHR

##### Overview

Many studies reported improvements in patient empowerment or the perception of improved empowerment with the adoption of PAEHRs. Empowerment included improvements in self-monitoring; medication management and self-management of conditions; increased engagement with health care providers, health screening, and care plans; improved understanding of and insight into one’s mental health; and feeling more prepared to engage with HCPs [37,50,52,54,57,59,67,68,73,76,77,90-95]. For example, patients in the study by Shin et al [66] reported that seeing their health record made them feel involved in the health care process, which was empowering. HCPs in the study by Kassam et al [54] thought PAEHRs helped dismantle power imbalances between patients and HCPs, fostering patient empowerment. Many of these studies were patients’ self-reports or clinicians’ perceptions of increased empowerment. However, Kipping et al [92] found that, when comparing patient portal users with nonusers, those who used the portal had increased appointment attendance, self-reported autonomy, and patient activation. Furthermore, in a randomized control trial, Druss et al [96,97] found that personal health record users had increased engagement with preventative health services.

PAEHRs could also support patients’ engagement with information by acting as a reference tool, supporting their preparation for and engagement in clinical encounters, reminding them of previous discussions with HCPs, and helping them keep track of their treatment [37,49,52,54,57-59,66,68,70,98]. PAEHRs may also improve patients’ knowledge of their health status, treatment, and medications [47,55,58,67,95]; increase the number of contacts with HCPs and the completion of follow-up assessments [94]; encourage patients to ask more questions about their treatment [62]; and help prepare them to discuss their treatment with an HCP [37,50,58]. The transparency promoted by the PAEHR, as discussed in subtheme 2.3, can also help patients track how their care is progressing [83]. Patients may also use the PAEHR to validate communications from their HCP and clarify information after a clinical encounter [52,66]. However, some patients were concerned that PAEHRs would replace face-to-face conversations with their HCPs [66].

In most studies that explored the rates of PAEHR adoption, the engagement of patients was low [84,85,92,99,100]. Connolly et al [101] found in a study of veterans with depression that only 21.9% (~669/3053) were registered for their My HealtheVet platform. HCPs across several studies also reported low PAEHR adoption and patient engagement with their PAEHR [49,52,57,93]. There were mixed findings on the demographic groups that were more likely to access their PAEHR. Connolly et al [101] found that patients with more severe symptoms were more likely to register for and download information from their patient portal. Some studies suggest that people with mental health conditions may be more likely to use a PAEHR [72,102], but this was not consistent across all studies [103]. PAEHR use may increase over time, with Onyeaka et al [12] finding in a national survey in the United States of people with depression and anxiety that patient portal use had increased from approximately a third to a half of respondents over 3 years. There were variable results regarding whether people with different diagnoses were more likely to use a PAEHR. Etingen et al [84] suggested that differences in use across diagnoses may be due to some diagnoses being more likely to be associated with other comorbidities, which may encourage or facilitate engagement with the PAEHR.

Interestingly, in studies that explored the functions of PAEHRs used by patients, viewing their health records is not always the primary use [12,84,101]. In many cases, other functions, such as medication refills and booking and viewing appointments, are used most [84]. Robotham [90] and Forchuk et al [98] also found that patients value visual representations of, and the ability to track changes in, their health information.

Only a few studies discussed patients documenting information in their PAEHR [37,55,66]. Patient-documented information is not a feature of all PAEHR systems, but some patients saw value in this functionality. For example, patients in the study by Shin et al [66] discussed how being able to report information in the PAEHR might help HCPs better understand their needs. In the study by Durocher et al [37], 1 participant thought it would be valuable if they could flag notes in the PAEHR and provide their interpretations of certain events.

##### Subtheme 6.1: Patients Need Support to Use Their PAEHR

Patients were more likely to access their PAEHR when HCPs spoke to them about accessing their PAEHR and when they understood its purpose [87]. However, in several studies, HCPs took a passive approach by letting patients figure out how to access and read their health record themselves [47,52,59,65]. In some studies, it appeared that not all HCPs had discussed the PAEHR with their patients; for example, 40% (82/205) of participants in the study by Dobscha et al [59] had not discussed it with their patients, and only a few participants reported having multiple conversations with patients about it. Similarly, in the study by Johansen et al [47], 50.8% (~169/332) of the HCPs surveyed reported that they informed patients that they could read their own EHR. In the study by Petersson et al [65] study, only 26.8% (180/671) of the HCPs encouraged patients to read their PAEHR, while 15.6% (105/671) initiated a discussion with patients about something in their PAEHR, and only 10% (67/670) used the PAEHR actively in treatment. This issue was also reflected in the study by Fagerlund [46], where patients could not recall being informed about the PAEHR and instead had heard about it through advertisements or media coverage. In addition, Leung et al [104] found that 50% (~51/103) of patients surveyed were aware that they could access their health records, while Bärkås et al [72] found that 64.77% (2028/3131) of patients in a national survey in Sweden had not been encouraged by anyone to read their PAEHR.

It is unclear why HCPs are not discussing the PAEHR with patients. Earlier themes might suggest that this finding is related to the perceived risks of PAEHR to patients or the lack of support for HCPs [53,59]. Some HCPs in the study by Zanaboni et al [49] suggested that the PAEHR is the patient’s tool, and therefore, the patient had to take the lead in using the PAEHR, rather than it being driven by the HCP. This perception of the PAEHR not being the HCP’s responsibility may be supported by a lack of training or information for HCPs [49].

Several studies implemented a PAEHR as part of an intervention or model of care, which appeared to lead to increased use by patients. For example, Weisner et al [105] found that patients in an intervention that involved six 45-minute sessions with a psychologist focused on patient education and activation had greater log-ins to a patient portal and checking of information in the portal than those not involved in the intervention. Kelly et al [93] found in a randomized controlled trial that patients were more likely to access a PAEHR when it was implemented alongside support from a peer navigator. Druss et al [97] also found that peer specialist involvement might contribute to higher PAEHR use.

Providing educational resources and support may help patients engage with their PAEHR. Kelly et al [93], when comparing low engagement in their study with higher engagement in the study by Druss et al [96], noted that the latter provided more intense resources and support to patients. Denneson et al [106] found in implementing a web-based education program for patients who accessed their health record on the internet that the training improved patients’ interactions and communication with HCPs and their own “activation.” The web-based program included information on what is included in their health record and how to use and communicate with clinicians about them.

##### Subtheme 6.2: PAEHRs Must Be Easy for Patients to Navigate, Access, and Understand

Several studies explored design requirements for PAEHRs and identified issues and opportunities to make it easier for patients to use their PAEHR [12,61,73,75,89,90,98,107,108]. Several studies identified login and account setup processes as an issue for patients, with recommendations that the process should be simplified, including addressing issues such as remembering passwords [12,75,83,89,98,109]. Patients also raised basic accessibility issues such as font size and colors [89,98,107] and navigation issues, including the number of links that patients had to navigate [79,107]. For example, in the study by van den Heuvel [91], patients who had dropped out of using an ePHR cited issues with the user friendliness as the main reason, along with the ePHR requiring too much work and effort. HCPs may need to support patients in navigating and understanding their PAEHRs, particularly patients with low health literacy or digital literacy, cognitive issues, and memory issues [68,71,107,108]. For HCPs to support patients, they need to be aware of what patients see in the record and how it functions for patients [58]. A lack of technology skills could be a barrier to patients engaging with their PAEHR [12,68], while some patients may also lack access to technology or the internet needed to use their PAEHR [12]. There is a risk that the benefits of PAEHRs may primarily benefit patients who are literate, educated, and affluent [10].

#### Theme 7: PAEHRs Raise New Concerns About Information Privacy and Security for HCPs, Patients, and Third Parties

##### Overview

Both patients and HCPs shared general concerns about privacy and security threats introduced by the PAEHR [49,52,57,62,68,76,104,107]. Some of these concerns were related more broadly to EHRs that multiple HCPs could access rather than the PAEHR itself [52,76]. For example, some patients in the studies by O’Neill et al [13] and Peck et al [76] raised concerns about their health record being available to other HCPs without their explicit consent. General concerns were also expressed about data hacking and breaches, but no participants reported experiences of these issues [10,62,68,76,77,79,104,107]. The most specific privacy threats identified were managing third-party data in the PAEHR and third-party access to the PAEHR, as outlined in the subsequent sections.

##### Subtheme 7.1: PAEHRs Pose Challenges to Managing Third-Party Data

An issue reported by HCPs was how to protect the privacy of third parties, such as family members, when documenting information they had provided in the health record information [49]. For example, HCPs might gather information about a patient from their family, which, when recorded in the PAEHR, would become visible to the patient. This risk might lead to HCPs reporting less information from family members or carers in the health record, which, as 1 participant in the study by Erlingsdóttir et al [57] said, will be a “big problem because it is important information that otherwise falls out of the system.” Another approach reported by participants in the study by Zanaboni et al [49] was to deidentify information provided by third parties.

##### Subtheme 7.2: PAEHRs Pose Challenges to Managing Third-Party Access to Patient Information

Both HCPs and patients raised concerns that PAEHRs could lead to third parties, such as family members, inappropriately accessing a patient’s health record [49,57,68,71]. Participants in the study by van Rijt et al [58] outlined the risk that patients could be influenced to share copies of their records or login details with family members who they may not want to have access to this information. There was also the broader issue of whether HCPs should record information assuming that the patient might give a third-party access to their PAEHR. For example, 1 participant in the study by Denneson et al [53] stated the following:

...someone said, “I smoked meth for 40 years and my wife doesn’t know.” And I was like, gosh, do I put this in the note? Because I don’t know if he is going to give his wife access to his notes and then see something that was delivered in confidence...

Such concerns might prompt HCPs to deny access altogether to certain information in the PAEHR to manage the risk of third-party access [49].

A few studies explored the potential benefits and issues with family members or carers being given access to PAEHRs [54,68,77,91,98,104]. One of the few studies that involved family and carers found that they thought it would be helpful to support the patient if they had access to the PAEHR [104]. Similarly, in the study by Leung et al [77], some patients reported that family or carer access could also be beneficial if it helped their family members or carers to better understand their health issues. However, in the same study, 43.7% (~43/103) of patients were interested in family members having access. The study by Schwarz et al [68] found that family access could enhance the exchange of information about the patient’s illness and help family members participate in the patient’s care. Van den Heuvel et al [91] reported that 23.1% (~9/39) of patients preferred informal caregivers to have access compared to 25.6% (10/39) finding it adverse. Similarly, Leung et al [104] found, in interviewing patients about a patient portal, that less than half of those interviewed were interested in family members having access to their records. There may also be scenarios where family members support the patient in using the PAEHR [110], which raises questions about how to ensure that the patient has the option to maintain privacy in those situations.

## Discussion

### Principal Findings

This scoping review explored how PAEHRs implemented in mental health care contexts change the information practices of HCPs and patients. We found that HCPs change their documentation practices in the presence of a PAEHR, including reducing the level of detail they document. HCPs changed their information practices because they were concerned that the PAEHR would negatively impact the therapeutic relationship and patient safety, and there was a lack of guidelines as to how these risks should be managed. There were clear benefits for patients who could use the PAEHR to understand and manage their care. However, patients may struggle to use the PAEHR without support and consideration of its ease of use.

The findings of this review suggest that HCPs perceive that PAEHRs change the purpose of the health record by requiring them to write with consideration of the patient as an audience rather than solely a clinical audience. It is a long-running issue that in an increasingly connected health care system, while technical interoperability between EHRs may increase, information is not necessarily written for multiple audiences [111]. The findings of this review suggest that digital health technologies that allow records created or updated by HCPs to be indiscriminately accessed by multiple types of users risk HCPs adopting information practices outside formal systems and processes, such as by creating separate records that are not officially recognized by the health service [49,112]. Omitting information from a patient’s health record not only fragments the record but also poses a risk to future care if information is lost or unavailable when needed. Potential technical advances, such as natural language processing and generative artificial intelligence, may solve this issue by supporting HCPs in translating their notes for different audiences [113-115].

HCPs in the included studies were concerned that PAEHRs would negatively impact the therapeutic relationship. The concern for the therapeutic relationship is not surprising, given it is a strong predictor of outcomes in mental health care [116]. HCPs and patients also thought that the PAEHR could strengthen the therapeutic relationship by contributing to transparency, communication, and trust. This finding aligns with evidence that mutual trust, demonstration of mutual respect, and shared decision-making were key components of a beneficial therapeutic relationship [117]. This finding may also explain why HCPs and patients saw benefits in the PAEHR facilitating communication about disagreements, as it helps build mutual trust [118]. However, both HCPs and patients expressed concerns that discussing what is documented in the PAEHR may add to workloads. A lack of time available for HCPs and patients to actively review and discuss the PAEHR may limit its use in strengthening the therapeutic relationship.

Risk to patient safety due to patients experiencing distress from reading their PAEHR was a key concern for both HCPs and patients. One of the key strategies used by HCPs to manage risk was to not document or limit the documentation of information that they perceived may be distressing for patients. This focus on risk is expected, given risk management has a prominent role in mental health care, as HCPs must consider and manage risks posed by patients to themselves or others [119]. One way HCPs manage risk is by avoiding situations that may involve heightened risks [119,120]. In this review, HCPs avoided risk by not documenting information they perceived could pose a risk to patients. This approach to avoiding risk may have been reinforced by a lack of guidelines for managing risk. HCPs are usually required to take a systematic approach to risk assessment, informed by organizational policies [121], which appeared to be lacking in relation to PAEHR adoption in the included studies. Evidence also suggests that there is value in involving patients in identifying and managing risks [122]. However, patient involvement in HCPs’ decisions to limit the documentation of information in the PAEHR appeared to be limited.

Patients wanted a greater breadth of information documented in their PAEHR, including strength-based and recovery-focused information. In contrast, HCPs managed the perceived risks of PAEHRs by reducing the depth of information they documented. These findings align with the growing literature on person-centered documentation, which seeks to consider the role patients can play in information documentation practices. Lyadhl et al [123] reflects that health record documentation practices are embedded in a biomedical model of care that prioritizes certain types of information to support communication between HCPs, rather than communication with patients. Person-centered documentation requires consideration of the patient’s goals, needs, resources, and narrative. “Collaborative documentation” is a model that supports HCPs and patients in working together to determine what should be documented during the clinical encounter [124,125]. It is a form of shared decision-making that seeks to improve the transparency of what is documented in the patient’s EHR [124]. Adopting a model such as collaborative documentation may address some of the concerns that PAEHRs will lead to disagreements about what is documented in the PAEHR.

PAEHRs can support new patient information practices, but there are barriers to patients using their PAEHR. PAEHR use by patients was low in the included studies, which is concerning given that recent studies in other health care settings have found generally high use rates [126,127]. The lower rates of PAEHR use in mental health care contexts may be partly explained by the slower adoption of PAEHRs in mental health care contexts, as the use of PAEHRs tends to increases over time [12,128]. It is also worth noting that many of the studies that considered use rates were conducted before the COVID-19 pandemic. The pandemic may have accelerated the adoption and use of PAEHRs due to the limits on in-person contact and the rapid adoption of telehealth [129].

Patient uptake of PAEHRs also relies on endorsement from HCPs [130]. However, our findings suggest that the lack of guidance for HCPs paired with the perceived risks to the therapeutic relationship and patient safety may act as a barrier to HCPs actively engaging patients in using their PAEHR. It was also identified that HCPs may not proactively engage certain groups of patients whom they perceive as at greater risk of negative outcomes from using their PAEHR. This finding may partly explain why, in some studies, patients with certain diagnoses were less likely to use their PAEHR. There is value in considering which patients may experience a negative response to a accessing their PAEHR, noting that some patients in the included studies did have negative experiences. Patient readiness measures, which have been found to be valid in measuring the likelihood of patients using health information technology [131,132], could form part of guidelines for HCPs to identify patients who need further support to use their PAEHR.

The time and resource pressures on HCPs are well documented, as are the impacts of these pressures on HCPs’ ability to engage with new digital health technologies [133,134]. In some mental health care services, other professionals could support patients to use their PAEHR, if HCPs are not provided with the time and resources to do this role. For example, peer workers are increasingly involved in mental health care services [135] and could play a role in supporting patients to use their PAEHR, as well as role-modeling how can be integrated into [136]. Similarly, care workers or “health care navigators” who in some health care systems support patients to move between health care services [137] may be well positioned to support patients in using their PAEHR. However, in all these cases, consideration will need to be given to how confidentiality of patient information is managed.

Managing third-party access to the PAEHR, particularly coercive access, was a key issue for HCPs but was not raised as a key issue by patients. It may be that patients at risk of having third parties inappropriately access their records were not participants in the included studies. The few studies that asked patients about family access found that they were uncomfortable with family members having access to their PAEHR. The issue of third-party access could be addressed through initiatives such as dynamic consent, where patients are given real-time control over managing their data through a personalized digital interface [138]. Australia’s national PAEHR, My Health Record, presents another approach for patients to manage third-party access by allowing them to nominate a trusted person to access their record and determine the level of access available to them [139]. Health care services could also provide kiosks for patients to access their PAEHR in the health care clinic if at-home access poses a risk of unauthorized third-party access. However, these approaches do not necessarily address the issue of third parties who may coerce patients into providing access to their PAEHR.

The findings of this review also raise questions as to how inequalities in patients’ resources and digital literacy may shape their interactions with a PAEHR. In the mental health care context, patients can experience socioeconomic disadvantages, which may limit their access to technology, meaning they may rely on smartphones or public computers [96,107,140]. PAEHRs should be designed to maintain privacy and accessibility, regardless of the location or type of device patients use to access them. There is a risk that the benefits of PAEHRs may disproportionately favor patients with more resources, rather than those facing the greatest health challenges [141,142]. These issues pose a more serious question about what the health system expects of patients when a PAEHR is available. Wynia and Dunn [143] outline 2 assumptions underlying PAEHRs: that more information for patients equals better decision-making and that patients should take greater responsibility for their health care. Lucivero [144] posits that PAEHRs may enroll patients into providing free labor for the health system where they act as intermediaries between HCPs by using their PAEHR to fill gaps in information. However, this role may be undermined if HCPs do not support patients to use their PAEHR, such as if HCPs are unwilling to act on information patients share with them, or if patients do not have the resources to fulfill this role.

### Comparison With Prior Work

The findings of this review align and extend the findings of recent reviews by Zhang et al [24] and Schwarz et al [9] in mental health care contexts. Some of the factors for successful implementation of patient portals identified by Zhang et al [24], such as training and resourcing, are also issues that we identified as contributing to some of the changes to HCPs’ information practices. Similar to the findings of Schwarz et al [9], we found that HCPs were more likely to express concerns about PAEHRs compared to patients. Our study extends the research into PAEHRs in mental health care contexts by framing the perceived risks of PAEHRs to the therapeutic relationship and patient safety as key factors in HCPs changing their information practice, further fueled by a lack of guidance and training. Several recent reviews of PAEHRs in other health care contexts also support the findings of this study. In a systematic review, Tapuria et al [31] found that patient portals had a range of benefits, such as reassuring patients and reducing anxiety, improving the physician-patient relationship, and improving patient outcomes. However, they also identified concerns regarding security, privacy and confidentiality, and patients experiencing anxiety with access to their records. In a recent systematic review of facilitators and barriers to electronic portal use, Powell [145] found that HCP encouragement is a key factor in both the initial and continued use of the PAEHR by patients. The findings of our review suggest that PAEHRs in mental health care contexts raise similar issues to their use in other health care contexts. However, we also found that that in mental health care contexts, HCPs still harbor significant concerns about how to protect the therapeutic relationship and patient safety—2 issues that have distinctive implications in the mental health care context.

### What Do the Findings Mean for Future Research?

Future research should consider how changes to HCP and patient information practices after the adoption of a PAEHR impacts clinical and patient experience outcomes. This study found that the use of PAEHRs led to changes in what is documented in the EHR and a potential “watering down” of information. Future research should explore the impact of these changes on collaborative care and clinical outcomes in the presence of a PAEHR. If these changes to information practices significantly impact the ability of other HCPs to provide appropriate care, it will strengthen the case for increased training, support, and guidance for how HCPs should document information in the presence of a PAEHR. It would also be valuable for more studies to explore the implementation and use of PAEHRs in practice and how HCP and patient use change over time, particularly given that use of PAEHRs tends to increase over time. There are very few studies that involved HCPs and patients in the process of designing a PAEHR. Given the findings of this review, there is a clear need for more studies that actively involve HCPs and patients in decisions about how PAEHRs are designed and implemented.

### Limitations

This review is limited in part due to the heterogeneity of the field, particularly the lack of common definitions of PAEHRs, which means that there may be studies that were missed in the search strategy. Furthermore, our search strategy did not include gray literature, meaning evaluations of some PAEHR systems that have not been published in academic journals may have been missed. This review was also limited by having only 1 author screen all the studies and extract the data for analysis. A pragmatic approach to screening was used, where only studies with unclear eligibility for inclusion were screened by a second author.

### Conclusions

This scoping review explored the impact of PAEHRs on information practices in mental health contexts. HCPs reported making various changes to their documentation practices to minimize the risk of PAEHRs harming the therapeutic relationship and patient safety. However, PAEHRs could also promote communication between HCPs and patients about what was being documented. PAEHRs also introduced new information practices for patients that allowed them to manage their health better and engage with health care services. PAEHRs are still considerably new in mental health care contexts, and patient adoption and use of established PAEHRs remain relatively low. Research is required on the impact of documentation changes on clinical outcomes and patient experience as well as approaches to improve adoption and use rates of PAEHRs. The findings of this study point to a lack of guidelines and support for HCPs and patients in the implementation of PAEHRs.

## Appendix

Multimedia Appendix 1 PRISMA-ScR (Preferred Reporting Items for Systematic Reviews and Meta-Analyses extension for Scoping Reviews) checklist.

Multimedia Appendix 2 Details of the included studies.

Multimedia Appendix 3 Illustrative quotes.

## Abbreviations

EHR: electronic health record
ePHR: electronic personal health record
HCP: health care professional
PAEHR: patient-accessible electronic health record
pEHR: personal electronic health record
PRISMA-ScR: Preferred Reporting Items for Systematic Reviews and Meta-Analyses extension for Scoping Reviews
