# Supplementary material for: Patient-Accessible Electronic Health Records and Information Practices in Mental Health Care Contexts: Scoping Review
Source: J Med Internet Res. 2025 Feb 7;27:e54973. doi: 10.2196/54973 (PMC11845895; doi:10.2196/54973)
Supplement: Multimedia Appendix 2 [file jmir_v27i1e54973_app2.docx]

**Appendix 2: Details of the included studies**

| **Reference** | **Country** | **Study Design** | **Research Method** | **Type of PAEHR [As categorized by research team]** | **Implementation Status** | **Main participants (patients, HCPs, both, other)** |
| --- | --- | --- | --- | --- | --- | --- |
| Abel et al. 2018 [1] | United States | Quantitative | Secondary data analysis | My HealtheVet | Implemented | Patients |
| Åkerstedt et al. 2018 [2] | Sweden | Quantitative | Survey | My Medical Record | Partial implementation, including if some participants were using or had access to the PAEHR | HCPs |
| Bärkås et al. 2021 [3] | Sweden | Qualitative | Document Analysis, interviews | Journalen | Implemented | HCPs |
| Bärkås et al. 2022 [4] | Multiple (Sweden, Estonia, Finland, Norway) | Qualitative | Focus groups / workshops | Patient Portal – General | Implemented | Other |
| Bärkås et al. 2023 [5] | Sweden | Quantitative | Survey | Journalen | Implemented | Patients |
| Durocher et al. 2023 [6] | Canada | Mixed Methods | Survey, interviews | MyCare (Cerner patient portal) | Implemented | Patients |
| Blease et al. 2021 [7] | Multiple (Canada, Estonia, Norway Sweden, UK, USA) | Qualitative | Survey | Patient Portal – General | Not applicable | Both |
| Blease et al. 2021 [8] | Multiple (Canada, Estonia, Norway, Sweden, UK, USA) | Quantitative | Survey | Patient Portal – General | Not applicable | Both |
| Blease et al. 2021 [9] | United States | Quantitative | Survey | Patient Portal – General | Implemented | Patients |
| Chimowitz et al. 2020 [10] | United States | Qualitative | Interviews and focus groups | Patient Portal – General | Partial implementation, including if some participants were using or had access to the PAEHR | HCPs |
| Connolly et al. 2020 [11] | United States | Quantitative | Secondary data analysis | MyHealtheVet | Implemented | Patients |
| Cromer et al. 2017 [12] | United States | Qualitative | Interviews | MyHealtheVet | Implemented | Patients |
| Denneson et al. 2017 [13] | United States | Qualitative | Interviews | MyHealtheVet | Implemented | HCPs |
| Denneson et al. 2018 [14] | United State | Quantitative | Survey | MyHealtheVet | Implemented | Patients |
| Denneson et al. 2019 [15] | United States | Quantitative | Intervention | MyHealtheVet | Implemented | Patients |
| Dobscha et al. 2016 [16] | United States | Quantitative | Survey | MyHealtheVet | Implemented | HCPs |
| Dobscha et al. 2018 [17] | United States | Quantitative | Survey and Secondary data analysis | MyHealtheVet | Implemented | Patients |
| Dobscha et al. 2019 [18] | United States | Quantitative | Intervention | MyHealtheVet | Implemented | HCPs |
| Druss et al. 2014 [19] | United States | Quantitative | Intervention | ePHR* – General | Implemented | Patients |
| Druss et al. 2020 [20] | United States | Quantitative | Intervention | ePHR – General | Implemented | Patients |
| Ennis et al. 2014 [21] | United Kingdom | Mixed Methods | Design | My Health Locker | PAEHR being designed as part of study | Both |
| Erlingsdottir et al. 2019 [22] | Sweden | Qualitative | Survey | Journalen | Pre-post | HCPs |
| Etingen et al. 2019 [23] | United States | Quantitative | Secondary data analysis | MyHealtheVet | Implemented | Patients |
| Eyasu et al. 2019 [24] | Canada | Qualitative | Design | Patient Portal – General | PAEHR being designed as part of study | Patients |
| Fagerlund et al. 2022 [25] | Norway | Qualitative | Interviews | Helsenorge.no | Implemented | Patients |
| Fisher et al. 2009 [26] | United Kingdom | Qualitative | Interviews and focus groups | Patient Portal – General | Implemented | Patients |
| Forchuk et al. 2013 [27] | Canada | Quantitative | Intervention | Lawson Smart Record | Implemented | Patients |
| Forchuk et al. 2014 [28] | Canada | Mixed Methods | Intervention | Lawson Smart Record | Implemented | Both |
| Forchuk et al. 2015 [29] | Canada | Mixed Methods | Intervention | Lawson Smart Record | Implemented | Patients |
| Forchuk et al. 2015 [30] | Canada | Qualitative | Intervention | Lawson Smart Record | Implemented | Patients |
| Gasteiger et al. 2020 [31] | New Zealand | Qualitative | Interviews and focus groups | Patient Portal – General | Soon-to-be implemented | Both |
| Hägglund et al. 2022 [32] | Sweden | Mixed Methods | Survey | Journalen | Implemented | Patients |
| Hilton et al. 2012 [33] [ | United States | Quantitative | Intervention | ePHR – General | Implemented | Patients |
| Hochwarter et al. 2022 [34] | Germany | Qualitative | Focus groups / workshops | Patient Portal – General | PAEHR being designed as part of study | Both |
| Johansen et a. 2019 [35] | Norway | Mixed Methods | Survey | Helsenorge.no | Implemented | HCPs |
| Jonnergård et al. 2021 [36] | Sweden | Mixed Methods | Survey and observation | Journalen | Soon-to-be implemented | HCPs |
| Kassam et al. 2022 [37] | Canada | Qualitative | Interviews | Patient Portal – General | Implemented | HCPs |
| Kelly et al. 2018 [38] | United States | Quantitative | Intervention | ePHR – General | Implemented | Patients |
| Kim et al. 2010 [39] | United States | Quantitative | Intervention | ePHR – General | Implemented | Patients |
| Kipping et al. 2016 [40] | Canada | Quantitative | Survey | Patient Portal – General | Implemented | Patients |
| Klein et al. 2018 [41] | United States | Quantitative | Survey | Patient Portal – General | Implemented | Patients |
| Kristiansen et al. 2019 [42] | Norway | Quantitative | Survey | Helsenorge.no | Implemented | HCPs |
| Leung et al. 2019 [43] | Canada | Mixed Methods | Survey | Patient Portal – General | Implements | Patients |
| Matthews et al. 2022 [44] | United States | Qualitative | Interviews | EPIC My Chart Portal | Implemented | Patients |
| Mayhew et al. 2018 [45] | Canada | Qualitative | Interviews | Patient Portal – General | Soon-to-be implemented | HCPs |
| O'Neill et al. 2019 [46] | United States | Mixed Methods | Interviews and surveys | Patient Portal – General | Implemented | Patients |
| Onyeaka et al. 2022 [47] | United States | Quantitative | Survey | Patient Portal – General | Implemented | Patients |
| Pecina et al. 2017 [48] | United States | Quantitative | Secondary data analysis | Patient Portal – General | Implemented | Patients |
| Peck et al. 2017 [49] | United States | Mixed Methods | Survey | Patient Portal – General | Implemented | Both |
| Petersson et al. 2018 [50] | Sweden | Quantitative | Survey | Journalen | Soon-to-be implemented | HCPs |
| Petersson et al. 2018 [51] | Sweden | Quantitative | Survey | Journalen | Implemented | HCPs |
| Petersson et al. 2021 [52] | Sweden | Qualitative | Interviews | Journalen | Implemented | HCPs |
| Pisciotta et al. 2019 [53] | United States | Qualitative | Interviews | MyHealtheVet | Implemented | Both |
| Raps et al. 2022 [54] | United States | Quantitative | Secondary data analysis | Patient Portal – General | Implemented | Patients |
| Robotham et al. 2015 [55] | United Kingdom | Mixed Methods | Intervention | MyHealthLocker | Implemented | Patients |
| Schwarz et al. 2023 [56] | Germany | Qualitative | Interviews | Patient Portal – General | Implemented | Patients |
| Shimada et al. 2014 [57] | United States | Quantitative | Secondary data analysis | MyHealtheVet | Implemented | Patients |
| Shin et al. 2023 [58] | Canada | Mixed Methods | Interview and survey | Patient Portal – General | Implemented | Patients |
| Strudwick et al. 2018 [59] | Canada | Mixed Methods | Survey | Patient Portal – General | Soon-to-be implemented | HCPs |
| Strudwick et al. 2020 [60] | Canada | Qualitative | Focus groups / workshops | Patient Portal – General | Unclear | Patients |
| Tsai et al. 2012 [61] | United States | Quantitative | Survey | MyHealtheVet | Implemented | Patients |
| Turvey et al. 2022 [62] | United States | Mixed Methods | Survey | Patient Portal – General | Partial implementation, including if some participants were using or had access to the PAEHR | Both |
| Van Den Heuvel et al. 2018 [63] | The Netherlands | Mixed Methods | Survey | ePHR – General | Implemented | Both |
| Van Rijt et al. 2021 [64] | The Netherlands | Qualitative | Interviews and focus groups | Patient Portal – General | Implemented | HCPs |
| Weisner et al. 2016 [65] | United States | Quantitative | Intervention | Patient Portal – General | Implemented | Patients |
| Zanaboni et al. 2022 [66] | Norway | Qualitative | Interviews | Helsenorge.no | Implemented | HCPs |

*ePHR: electronic personal health record.

**Reference**

[1] Abel EA, Shimada SL, Wang K, Ramsey C, Skanderson M, Erdos J, Godleski L, Houston TK, Brandt CA. Dual use of a patient portal and clinical video telehealth by veterans with mental health diagnoses: retrospective, cross-sectional analysis. J Med Internet Res 2018 Nov 07; 20(11):e11350

[2] Åkerstedt US, Cajander Å, Moll J, Ålander T. On threats and violence for staff and patient accessible electronic health records. Cogent Psychol 2018 Sept 27; 5(1):1518967

[3] Bärkås A, Scandurra I, Rexhepi H, Blease C, Cajander Å, Hägglund M. Patients' access to their psychiatric notes: current policies and practices in Sweden. Int J Environ Res Public Health 2021 Aug 30; 18(17):9140

[4] Bärkås A, Hägglund M, Moll J, Cajander Å, Rexhepi H, Hörhammer I, Blease C, Scandurra I. Patients' access to their psychiatric records - a comparison of four countries. Stud Health Technol Inform 2022 May 25; 294:510-4

[5] Bärkås A, Kharko A, Blease C, Cajander Å, Johansen Fagerlund A, Huvila I, Johansen MA, Kane B, Kujala S, Moll J, Rexhepi H, Scandurra I, Wang B, Hägglund M. Errors, omissions, and offenses in the health record of mental health care patients: results from a nationwide survey in Sweden. J Med Internet Res 2023 Nov 03; 25:e47841

[6] Durocher K, Shin HD, Lo B, Chen S, Ma C, Strudwick G. Understanding the role of patient portals in fostering interprofessional collaboration within mental health care settings: mixed methods study. JMIR Hum Factors 2023 Jul 19; 10:e44747

[7] Blease C, Torous J, Kharko A, DesRoches CM, Harcourt K, O'Neill S, Salmi L, Wachenheim D, Hägglund M. Preparing patients and clinicians for open notes in mental health: qualitative inquiry of international experts. JMIR Ment Health 2021 Apr 16; 8(4):e27397

[8] Blease C, Kharko A, Hägglund M, O'Neill S, Wachenheim D, Salmi L, Harcourt K, Locher C, DesRoches CM, Torous J. The benefits and harms of open notes in mental health: a Delphi survey of international experts. PLoS One 2021 Oct 13; 16(10):e0258056

[9] Blease C, Dong Z, Torous J, Walker J, Hägglund M, DesRoches CM. Association of patients reading clinical notes with perception of medication adherence among persons with serious mental illness. JAMA Netw Open 2021 Mar 01; 4(3):e212823

[10] Chimowitz H, O'Neill S, Leveille S, Welch K, Walker J. Sharing psychotherapy notes with patients: therapists' attitudes and experiences. Soc Work 2020 Apr 01; 65(2):159-68

[11] Connolly SL, Etingen B, Shimada SL, Hogan TP, Nazi K, Stroupe K, Smith BM. Patient portal use among veterans with depression: associations with symptom severity and demographic characteristics. J Affect Disord 2020 Oct 01; 275:255-9

[12] Cromer R, Denneson LM, Pisciotta M, Williams H, Woods S, Dobscha SK. Trust in mental health clinicians among patients who access clinical notes online. Psychiatr Serv 2017 May 01; 68(5):520-3

[13] Denneson LM, Cromer R, Williams HB, Pisciotta M, Dobscha SK. A qualitative analysis of how online access to mental health notes is changing clinician perceptions of power and the therapeutic relationship. J Med Internet Res 2017 Jun 14; 19(6):e208

[14] Denneson LM, Chen JI, Pisciotta M, Tuepker A, Dobscha SK. Patients' positive and negative responses to reading mental health clinical notes online. Psychiatr Serv 2018 May 01; 69(5):593-6

[15] Denneson LM, Pisciotta M, Hooker ER, Trevino A, Dobscha SK. Impacts of a web-based educational program for veterans who read their mental health notes online. J Am Med Inform Assoc 2019 Jan 01; 26(1):3-8

[16] Dobscha SK, Denneson LM, Jacobson LE, Williams HB, Cromer R, Woods S. VA mental health clinician experiences and attitudes toward OpenNotes. Gen Hosp Psychiatry 2016; 38:89-93

[17] Dobscha SK, Denneson LM, Pisciotta MK, Bourne DS, Chen JI, Philipps-Moses D, Woods SS. Predictors of viewing progress notes among users of VA's electronic health portal who receive mental health care. JAMIA Open 2018 Jul; 1(1):122-7

[18] Dobscha SK, Kenyon EA, Pisciotta MK, Niederhausen M, Woods S, Denneson LM. Impacts of a web-based course on mental health clinicians' attitudes and communication behaviors related to use of OpenNotes. Psychiatr Serv 2019 Jun 01; 70(6):474-9

[19] Druss BG, Ji X, Glick G, von Esenwein SA. Randomized trial of an electronic personal health record for patients with serious mental illnesses. Am J Psychiatry 2014 Mar; 171(3):360-8

[20] Druss BG, Li J, Tapscott S, Lally CA. Randomized trial of a mobile personal health record for behavioral health homes. Psychiatr Serv 2020 Aug 01; 71(8):803-9

[21] Ennis L, Robotham D, Denis M, Pandit N, Newton D, Rose D, Wykes T. Collaborative development of an electronic Personal Health Record for people with severe and enduring mental health problems. BMC Psychiatry 2014 Nov 18; 14:305

[22] Erlingsdóttir G, Petersson L, Jonnergård K. A theoretical twist on the transparency of open notes: qualitative analysis of health care professionals' free-text answers. J Med Internet Res 2019 Sept 25; 21(9):e14347

[23] Etingen B, Hogan TP, Martinez RN, Shimada S, Stroupe K, Nazi K, Connolly SL, Lipschitz J, Weaver FM, Smith B. How do patients with mental health diagnoses use online patient portals? An observational analysis from the Veterans Health Administration. Adm Policy Ment Health 2019 Sept; 46(5):596-608

[24] Eyasu T, Leung K, Strudwick G. Guiding improvements in user experience: results of a mental health patient portal user interface assessment. Stud Health Technol Inform 2019; 257:110-4

[25] Fagerlund AJ, Kristiansen E, Simonsen RA. Experiences from using patient accessible electronic health records - a qualitative study within Sámi mental health patients in Norway. Int J Circumpolar Health 2022 Dec 17; 81(1):2025682

[26] Fisher B, Bhavnani V, Winfield M. How patients use access to their full health records: a qualitative study of patients in general practice. J R Soc Med 2009 Dec; 102(12):539-44

[27] Forchuk C, Rudnick A, Hoch J, Donelle L, Godin M, Osoka W, Campbell R, Rasmussen D, Edwards B, Osuch E, Norman R, Vingillis E, Mitchell B, Reiss J, Petrenko M, Corring D, McKillop M. Mental Health Engagement Network (MHEN). Int J Adv Life Sci 2013; 5(1&2):1-10

[28] Forchuk C, Rudnick A, Hoch J, Donelle L, Campbell R, Osaka W, Edwards B, Osuch E, Norman R, Vingilis E, Mitchell B, Reiss J, Corring D, Petrenko M, Godin M, Reed J, McKillop M. Mental health engagement network: innovating community-based mental healthcare. J Gen Pract 2014; 02(01):0

[29] Forchuk C, Donelle L, Ethridge P, Warner L. Client perceptions of the mental health engagement network: a secondary analysis of an intervention using smartphones and desktop devices for individuals experiencing mood or psychotic disorders in Canada. JMIR Ment Health 2015; 2(1):e1

[30] Forchuk C, Reiss JP, O'Regan T, Ethridge P, Donelle L, Rudnick A. Client perceptions of the mental health engagement network: a qualitative analysis of an electronic personal health record. BMC Psychiatry 2015 Oct 14; 15:250

[31] Gasteiger N, Fleming T, Day K. Converging perspectives of providers and student users on extending a patient portal into a university-based mental health service: a qualitative study. Internet Interv 2020 Mar; 19:100304

[32] Hägglund M, Scandurra I. Usability of the Swedish accessible electronic health record: qualitative survey study. JMIR Hum Factors 2022 Jun 23; 9(2):e37192

[33] Hilton JF, Barkoff L, Chang O, Halperin L, Ratanawongsa N, Sarkar U, Leykin Y, Munoz RF, Thom DH, Kahn JS. A cross-sectional study of barriers to personal health record use among patients attending a safety-net clinic. PloS one 2012 Feb 20; 7(20): e31888.

[34] Hochwarter S, Fehler G, Muente C, Eisenmann Y, Heinze M, Hibsch C, Schwarz J. Design of a patient-accessible electronic health record system in mental health. Stud Health Technol Inform 2022 May 25; 294:583-4

[35] Johansen MA, Kummervold PE, Sørensen T, Zanaboni P. Health professionals' experience with patients accessing their electronic health records: results from an online survey. Stud Health Technol Inform 2019 Aug 21; 264:504-8

[36] Jonnergård K, Petersson L, Erlingsdóttir G. Communicating the implementation of open notes to health care professionals: mixed methods study. JMIR Med Inform 2021 Aug 16; 9(8):e22391

[37] Kassam I, Shin HD, Durocher K, Lo B, Shen N, Mehta R, Sockalingam S, Wiljer D, Gratzer D, Sequeira L, Strudwick G. "I think it's something that we should lean in to": the use of OpenNotes in Canadian psychiatric care contexts by clinicians. Digit Health 2022; 8:20552076221144106

[38] Kelly EL, Braslow JT, Brekke JS. Using electronic health records to enhance a peer health navigator intervention: a randomized pilot test for individuals with serious mental illness and housing instability. Community Ment Health J 2018 Nov 3; 54(8):1172-9

[39] Kim EH, Kim Y. Digital divide: use of electronic personal health record by different population groups. Annu Int Conf IEEE Eng Med Biol Soc 2010; 2010:1759-62

[40] Kipping S, Stuckey MI, Hernandez A, Nguyen T, Riahi S. A web-based patient portal for mental health care: benefits evaluation. J Med Internet Res 2016 Nov 16; 18(11):e294

[41] Klein JW, Peacock S, Tsui JI, O'Neill SF, DesRoches CM, Elmore JG. Perceptions of primary care notes by patients with mental health diagnoses. Ann Fam Med 2018 Jul; 16(4):343-5

[42] Kristiansen E, Johansen M, Zanaboni P. Healthcare personnels’ experience with patients’ online access to electronic health records: differences between professions, regions, and somatic and psychiatric healthcare. In: Proceedings of the 17th Scandinavian Conference on Health Informatics. 2019. Presented at: SHI 2019; November 12-13, 2019; Oslo, Norway

[43] Leung K, Clark C, Sakal M, Friesen M, Strudwick G. Patient and family member readiness, needs, and perceptions of a mental health patient portal: a mixed methods study. Stud Health Technol Inform 2019; 257:266-70

[44] Matthews EB, Savoy M, Paranjape A, Washington D, Hackney T, Galis D, Zisman-Ilani Y. Acceptability of health information exchange and patient portal use in depression care among underrepresented patients. J Gen Intern Med 2022 Nov; 37(15):3947-55

[45] Mayhew C, Strudwick G, Waddell J. Clinical nurse specialists’ perceptions of a mental health patient portal. Clin Nurse Spec 2018; 32(6):313-22

[46] O'Neill S, Chimowitz H, Leveille S, Walker J. Embracing the new age of transparency: mental health patients reading their psychotherapy notes online. J Ment Health 2019 Oct; 28(5):527-35

[47] Onyeaka H, Ajayi KV, Muoghalu C, Eseaton PO, Azuike CO, Anugwom G, Oladunjoye F, Aneni K, Firth J, Torous J. Access to online patient portals among individuals with depression and anxiety. Psychiatry Res Commun 2022 Dec; 2(4):100073

[48] Pecina J, North F, Williams MD, Angstman KB. Use of an on-line patient portal in a depression collaborative care management program. J Affect Disord 2017 Jan 15; 208:1-5

[49] Peck P, Torous J, Shanahan M, Fossa A, Greenberg W. Patient access to electronic psychiatric records: a pilot study. Health Policy Technol 2017 Sept; 6(3):309-15

[50] Petersson L, Erlingsdóttir G. Open notes in Swedish psychiatric care (part 1): survey among psychiatric care professionals. JMIR Ment Health 2018 Feb 02; 5(1):e11

[51] Petersson L, Erlingsdóttir G. Open notes in Swedish psychiatric care (part 2): survey among psychiatric care professionals. JMIR Ment Health 2018 Jun 21; 5(2):e10521

[52] Petersson J, Backman C. Off the record: the invisibility work of doctors in a patient-accessible electronic health record information service. Sociol Health Illn 2021 Jun; 43(5):1270-85

[53] Pisciotta M, Denneson LM, Williams HB, Woods S, Tuepker A, Dobscha SK. Providing mental health care in the context of online mental health notes: advice from patients and mental health clinicians. J Ment Health 2019 Feb; 28(1):64-70

[54] Raps SJ, Chen D, Bakken S, Caban J, Engler MB. Baseline eHealth behaviors of service members: a retrospective, cross-sectional analysis of patient portal use before the pandemic. Mil Med 2023 Jul 22; 188(7-8):e2598-605

[55] Robotham D, Mayhew M, Rose D, Wykes T. Electronic personal health records for people with severe mental illness; a feasibility study. BMC Psychiatry 2015 Aug 06; 15:192

[56] Schwarz J, Meier-Diedrich E, Neumann K, Heinze M, Eisenmann Y, Thoma S. Reasons for acceptance or rejection of online record access among patients affected by a severe mental illness: mixed methods study. JMIR Ment Health 2024 Feb 05; 11:e51126

[57] Shimada SL, Brandt CA, Feng H, McInnes DK, Rao SR, Rothendler JA, Haggstrom DA, Abel EA, Cioffari LS, Houston TK. Personal health record reach in the Veterans Health Administration: a cross-sectional analysis. J Med Internet Res 2014 Dec 12; 16(12):e272

[58] Shin HD, Durocher K, Lo B, Chen S, Ma C, Wiljer D, Strudwick G. Impact of a mental health patient portal on patients’ views of compassion: a mixed-methods study. BMC Digit Health 2023 Jan 24; 1(1):0

[59] Strudwick G, Clark C, Sanches M, Strauss J. Predictors of mental health professionals' perceptions of patient portals. AMIA Annu Symp Proc 2018; 2018:989-97

[60] Strudwick G, Booth RG, McLean D, Leung K, Rossetti S, McCann M, Strauss J. Identifying indicators of meaningful patient portal use by psychiatric populations. Inform Health Soc Care 2020 Oct 01; 45(4):396-409

[61] Tsai J, Rosenheck RA. Use of the internet and an online personal health record system by US veterans: comparison of Veterans Affairs mental health service users and other veterans nationally. J Am Med Inform Assoc 2012; 19(6):1089-94

[62] Turvey CL, Fuhrmeister LA, Klein DM, Moeckli J, Howren MB, Chasco EE. Patient and provider experience of electronic patient portals and secure messaging in mental health treatment. Telemed J E Health 2022 Feb; 28(2):189-98

[63] van den Heuvel SC, Meije D, Regeer EJ, Sinnema H, Riemersma RF, Kupka RW. The user experiences and clinical outcomes of an online personal health record to support self-management of bipolar disorder: a pretest-posttest pilot study. J Affect Disord 2018 Oct 01; 238:261-8

[64] van Rijt AM, Hulter P, Weggelaar-Jansen AM, Ahaus K, Pluut B. Mental health care professionals' appraisal of patients' use of web-based access to their electronic health record: qualitative study. J Med Internet Res 2021 Aug 27; 23(8):e28045

[65] Weisner CM, Chi FW, Lu Y, Ross TB, Wood SB, Hinman A, Pating D, Satre D, Sterling SA. Examination of the effects of an intervention aiming to link patients receiving addiction treatment with health care: the LINKAGE clinical trial. JAMA Psychiatry 2016 Aug 01; 73(8):804-14

[66] Zanaboni P, Kristiansen E, Lintvedt O, Wynn R, Johansen MA, Sørensen T, Fagerlund AJ. Impact on patient-provider relationship and documentation practices when mental health patients access their electronic health records online: a qualitative study among health professionals in an outpatient setting. BMC Psychiatry 2022 Jul 28; 22(1):508
