# Supplementary material for: Patient-Accessible Electronic Health Records and Information Practices in Mental Health Care Contexts: Scoping Review
Source: J Med Internet Res. 2025 Feb 7;27:e54973. doi: 10.2196/54973 (PMC11845895; doi:10.2196/54973)
Supplement: Multimedia Appendix 3 [file jmir_v27i1e54973_app3.docx]

**Appendix 3: Illustrative Quotes**

| Theme | Sub-theme |
| --- | --- |
| Theme 1: Patient accessible electronic health records (PAEHRs) change the purpose of the health record | “I’m thoughtful about my notes, but the idea of having to think about one more audience when we’re already writing for insurance companies, we’re writing for the physicians, and I also want to make my notes somewhat meaningful . . . it just felt burdensome.” – HCP [1] |
|  | “Yes it definitely has changed. There was a definite time we used to get a… message saying ‘can this person read their own record?’ In an ideal world the person would come to us and we could say, ‘Please, you may find words or language in there that is confusing to you; don’t hesitate to give us a call, we’d love to talk this over with you if you have anything - some feelings may come up for you reading what clinicians have written about you.’” - HCP [2] |
| Theme 2: Health care professionals (HCPs) change their information practices to protect the therapeutic relationship and patient safety | “I see great danger in that the pact with the patient will be broken rather than strengthened, and that much of the work after that will be focused on the notes rather than on the rehabilitation/recovery from the psychiatric diagnosis.” – HCP [3] |
| Subtheme 2.1: PAEHRs introduce risks to the therapeutic relationship | “The therapist only said supportive things to me but the note seemed judgmental in a negative way. After reading it, I felt badly, like she didn’t like me as much as I had thought.” - Patient [4] |
| Subtheme 2.2: PAEHRs introduce risks to patient safety | “Then again, for me, the onus is on us. We’re the ones who are responsible for creating safety. I think that’s a big part of this. If OpenNotes were to trigger somebody or create a safety issue, it’s still on us to do our best to resolve it in a safe way. It shouldn’t be on the person who is sick or war-torn to navigate it.” - HCP [2] |
| Subtheme 2.3: Trust, transparency and accuracy of information can mitigate the risks posed by PAEHRs to the therapeutic relationship and patient safety | “I think being open and saying, ‘Well this is what I’m going to put in your note’ rather than having it come as a surprise. Because once I discovered that…and maybe they have a different perspective on it than I do. Yes, they have the doctorate, but I’m the person living through it, so who has the right perspective?” – Patient [5] |
|  | «I think that being more open can be positive for the cooperation with the patient and makes it easier to develop an alliance – HCP [6] |
| Subtheme 2.4: PAEHRs offer opportunities to enhance accountability and communication between HCPs and patients about what is documented | “Say somebody’s at home ...and there’s something in the note that they feel is not accurate or is disturbing to them ...I feel like in the moment, that could be upsetting for somebody, and that also goes back to the provider though. That’s an indication [of ] more communication” – HCP [1] |
|  | A problem I experience is that healthcare doesn’t seem to document properly in the record. I have a feeling that there is a real record...but I can only see part of it. Or the documentation is so poor that what I see is all there is. If that’s the case, it worries me.” – Patient [7] |
| Theme 3: PAEHRs disrupt HCP’s documentation practices | “I wrote a note in the medical record in a certain way, but maybe the other person (the patient) experienced a different conversation.” – HCP [8] |
|  | “…suppose you have seen or recognized something, and you did not want to write it down for whatever reason, but it does have an influence on a future course of the treatment, or possibly a crisis situation, and you say: “well, I did see or spot that earlier on” – HCP [8] |
| Sub-theme 3.1: HCPs document less detailed information in PAEHRs, particularly relating to sensitive information | “How have I adapted? My notes are a lot less detailed now, here. I always have to kind of couch what I’m saying. There’s much less detail, much less frankness in my notes now” – HCP [9] |
| Sub-theme 3.2: PAEHRs require HCPs to document information that patients will understand | “Try to limit jargon and instead maybe use language that is more, not dumbing down, but more understandable: more descriptors, more explanation, more rational.” HCP [5] |
| Sub-theme 3.3: PAEHRs require HCPs to take a person-centered approach when documenting potentially subjective information | “Let’s assume I see someone who looks dirty or with poor hygiene, then I have a hard time writing that down.” – HPC [8] |
|  | “I am less likely to use terms that are clinically accurate but may be read as pejorative” – HCP [10] |
|  | Yes, there were things that were important to me, but I could not find them in the journal. I especially remember being in one institution, where we talked about my large family and many relatives. This was not mentioned in the journal – Patient [11] |
| Sub-Theme: 3.4 HCPs limit the documentation of uncertain information in PAEHRs | “ The notes becomes a poorer quality work document for me as a doctor. I can no longer write down all the tentative diagnoses and hypotheses.” [3] |
| Sub-Theme 3.5 HCPs may omit content or restrict access to PAEHRs | “I think it is not right that patients in psychiatry should have access to their journal. In fear of writing something "offensive" I think many therapists unfortunately have to do double-journal entries. Which again is vulnerable to getting lost.” – HCP [12] |
|  | “I would use Journalen more if the whole record was included. The fact that the psychiatric notes are not included makes me feel discriminated and fragmented as a person. Body and mind affect each other and somatic care needs to consider what happens in psychiatry and vice versa.” – Patient [7] |
| Theme 4: PAEHRs introduce changes to HCPs’ information workflows | If I have some hypotheses, I try to present them to the patient first, and eventually write them down afterwards – HCP [6] |
| Theme 5: HCPs require tailored training, education, and guidelines on documenting information in a PAEHR. | “I would appreciate some clarity on who the audience is, on who I’m writing for. And I think in general, training in this more recovery-oriented and strengths-oriented treatment in general. Moving away from thinking about things, like in the medical model, in terms of problems and thinking about things more as this being a collaborative relationship with their clients.” – HCP [2] |
| Theme 6: Patients are empowered with new information practices if they are supported to use their PAEHR. | “You really want to use that 10 minutes to maximum effect so I find I have to prep up and refresh my memory . so when I hit the doctor, I am up and running and he knows that I have already read the thing and I put my points about the data in front of him.” – Patient [13] |
|  | “The comment that I had about the notes is it would have been nice for me to be able to flag certain things. I had been at an inpatient facility and one of the nurses there had given an account of events about how something had occurred. I would have really appreciated the opportunity to flag that and give my interpretation, because in the portal there was only one... it was great to see what was written, but there was only one side to it.” – Patient [14] |
| Sub-theme 6.1: Patients need support to use their PAEHR | “I think of all therapy as adult to adult. If [patients are] interested somehow . . . they’ll figure it out if they want to look at [their notes]” – HCP [1] |
| Sub-theme 6.2: PAEHRs must Be Easy for Patients to Navigate, Access, and Understand | "I think the user interface is a bit cumbersome and it is not always clear where you can find the information you’re looking for. I usually use my mobile phone and the user interface is poorly adapted for mobile use. Perhaps a special mobile app would be good.” - Patient[7] |
| Theme 7: PAEHRs introduce new information privacy and security concerns for HCPs, patients, and third parties. | “I strongly oppose to have these notes on [the patient portal] available to anyone who has authorization to read them. It is a violation of patients’ rights to have private and very personal conversation opened up for anyone to see. This is not my idea of therapy and because of this reason I quit seeing the therapist” - Patient [4] |
| Sub-theme 7.1: PAEHRs pose challenges to managing third-party data | “I’m less candid about the information that relatives provide. That has sometimes been a big problem because it is important information that otherwise falls out of the system.” - HCP [3] |
| Sub-theme 7.2: PAEHRs pose challenges to managing third-party access to patient information | “People who live in coercive relationships, maltreated, can be forced to log in” – HCP [6] |

**References**

[1] Chimowitz H, O'Neill S, Leveille S, Welch K, Walker J. Sharing psychotherapy notes with patients: therapists' attitudes and experiences. Soc Work 2020 Apr 01; 65(2):159-68

[2] Cromer R, Denneson LM, Pisciotta M, Williams H, Woods S, Dobscha SK. Trust in mental health clinicians among patients who access clinical notes online. Psychiatr Serv 2017 May 01; 68(5):520-3

[3] Erlingsdóttir G, Petersson L, Jonnergård K. A theoretical twist on the transparency of open notes: qualitative analysis of health care professionals' free-text answers. J Med Internet Res 2019 Sept 25; 21(9):e14347

[4] O'Neill S, Chimowitz H, Leveille S, Walker J. Embracing the new age of transparency: mental health patients reading their psychotherapy notes online. J Ment Health 2019 Oct; 28(5):527-35

[5] Pisciotta M, Denneson LM, Williams HB, Woods S, Tuepker A, Dobscha SK. Providing mental health care in the context of online mental health notes: advice from patients and mental health clinicians. J Ment Health 2019 Feb; 28(1):64-70

[6] Zanaboni P, Kristiansen E, Lintvedt O, Wynn R, Johansen MA, Sørensen T, Fagerlund AJ. Impact on patient-provider relationship and documentation practices when mental health patients access their electronic health records online: a qualitative study among health professionals in an outpatient setting. BMC Psychiatry 2022 Jul 28; 22(1):508

[7] Hägglund M, Scandurra I. Usability of the Swedish accessible electronic health record: qualitative survey study. JMIR Hum Factors 2022 Jun 23; 9(2):e37192

[8] van Rijt AM, Hulter P, Weggelaar-Jansen AM, Ahaus K, Pluut B. Mental health care professionals' appraisal of patients' use of web-based access to their electronic health record: qualitative study. J Med Internet Res 2021 Aug 27; 23(8):e28045

[9] Denneson LM, Cromer R, Williams HB, Pisciotta M, Dobscha SK. A qualitative analysis of how online access to mental health notes is changing clinician perceptions of power and the therapeutic relationship. J Med Internet Res 2017 Jun 14; 19(6):e208

[10] Peck P, Torous J, Shanahan M, Fossa A, Greenberg W. Patient access to electronic psychiatric records: a pilot study. Health Policy Technol 2017 Sept; 6(3):309-15

[11] Fagerlund AJ, Kristiansen E, Simonsen RA. Experiences from using patient accessible electronic health records - a qualitative study within Sámi mental health patients in Norway. Int J Circumpolar Health 2022 Dec 17; 81(1):2025682

[12] Johansen MA, Kummervold PE, Sørensen T, Zanaboni P. Health professionals' experience with patients accessing their electronic health records: results from an online survey. Stud Health Technol Inform 2019 Aug 21; 264:504-8

[13] Fisher B, Bhavnani V, Winfield M. How patients use access to their full health records: a qualitative study of patients in general practice. J R Soc Med 2009 Dec; 102(12):539-44

[14] Durocher K, Shin HD, Lo B, Chen S, Ma C, Strudwick G. Understanding the role of patient portals in fostering interprofessional collaboration within mental health care settings: mixed methods study. JMIR Hum Factors 2023 Jul 19; 10:e44747
